# AstroGreen transgenic mouse illuminates the trafficking of astrocyte-derived extracellular vesicles

**DOI:** 10.1016/j.mcn.2025.104051

**Published:** 2025-10-08

**Authors:** Lisa Nieland, Edwina Abou Haidar, David Rufino-Ramos, Shilpa Prabhakar, Youssef Samaha, Koen Breyne, Francis K. Fordjour, Saumya Das, Marike L.D. Broekman, Stephen Gould, Xandra O. Breakefield, Erik R. Abels

**Affiliations:** a Department of Molecular Neurogenetics Unit, Massachusetts General Hospital, 13^th^ Street, Building 149, Charlestown, MA, 02129, USA; b Department of Neurosurgery, Leiden University Medical Center, Leiden, 2300 RC, the Netherlands; c CNC—Center for Neuroscience and Cell Biology, University of Coimbra, Rua Larga, Coimbra, 3004-504, Portugal; d Center for Genomic Medicine, Massachusetts General Hospital, Boston, MA, 02114, USA; e Department of Pathology, Massachusetts General Hospital, Boston, MA, 02114, USA; f Department of Pathology, Harvard Medical School, Boston, MA, 02115, USA; g Department of Biological Chemistry, Johns Hopkins University, Baltimore, MD, 21218, USA; h Cardiovascular Research Center, Massachusetts General Hospital, Harvard Medical School, Boston, MA, 02114, USA; i Department of Neurosurgery, Haaglanden Medical Center, The Hague, 2512 VA, the Netherlands; j Department of Cell and Chemical Biology, Leiden University Medical Center, Leiden, 2300 RC, the Netherlands.

**Keywords:** Glia, Astrocytes, Extracellular vesicles, Transgenic mouse model, Cre reporter

## Abstract

Astrocytes interact with neighboring cells by releasing extracellular vesicles (EVs). Tools to study astrocyte EV-mediated communication with other brain cells *in vivo* are essential. In this study, we crossed the Exomap1 transgenic mouse expressing Cre-activated human-specific CD81 (HsCD81) fused to the fluorescent protein mNeonGreen (HsCD81mNG), to a transgenic mouse expressing Cre under the astrocyte-expressing GFAP promoter resulting in *Exomap1*::*Gfap-Cre* mice, referred to here as AstroGreen. We characterized HsCD81mNG-expressing astrocytes and shedded EVs loaded with HsCD81mNG and Cre, both *in vitro* and in mouse brains. Using this model, we show that HsCD81mNG can be used to track EV content, production, and functional Cre transfer *in vitro* and in the brain, allowing evaluation of the interaction of astrocytes with neighboring cells mediated by EVs. We anticipate that this model will improve our understanding of astrocytes transferring EVs within their surroundings during normal physiological processes and in the context of neuropathological conditions.

## 1. Introduction

Astrocytes are the most abundant glial cell type in the central nervous system (CNS) (Sofroniew and Vinters, 2010). Initially, astrocytes were described to solely support neuronal networks (Kimelberg, 2004; Gonzalez-Perez et al., 2015). However, growing evidence has shown that astrocytes are involved in a broad range of functions in both healthy and diseased brains (Kimelberg and Nedergaard, 2010; Brandao et al., 2019; Parpura and Verkhratsky, 2012; Perelroizen et al., 2022). Astrocytes express five types of intermediate filaments including glial fibrillary acidic protein (GFAP); vimentin; nestin; synemin; and lamins (Potokar et al., 2020). High levels of intermediate filament protein GFAP, contributes to the distinct cytoarchitecture and mechanical strength of astrocytes (Middeldorp and Hol, 2011). In addition to being a reliable astrocyte marker protein, GFAP expression increases in response to CNS injury, infections, and diseases, resulting in thickening and elongation of astrocytic processes during activation of astrocytes, termed reactive astrogliosis (McKeon and Benarroch, 2018; Escartin et al., 2021; Sofroniew, 2020; Hol and Pekny, 2015; Pekny and Pekna, 2004). The full extent of the implications of astrocyte activation has yet to be elucidated, but evidence increasingly indicates that reactive astrocytes maintain complex interactions with different cell types in the CNS through a variety of biomolecular cell-to-cell interactions, including gap-junction-mediated exchange of small molecules, nanotube-mediated exchange of larger molecules, and indirectly through the secretion of cytokines, neurotrophins, gliotransmitters and extracellular vesicles (EVs) (Gurke et al., 2008; Theodoric et al., 2012; Ding et al., 2021).

There are many subtypes of EVs identified, based on their size, content, function, and biogenesis (Doyle and Wang, 2019; Pegtel and Gould, 2019). EVs are a heterogeneous population of lipid bilayer-membrane bound nanosized particles, including small EVs (sEVs; <200 nm in diameter) and large EVs (lEVs; >200 nm in diameter) (Thery et al., 2018; Welsh et al., 2024). EVs are shed by all cell types and originate mainly by a combination of direct budding from the plasma membrane or mitochondrial membranes, as well as by vesicle budding into endosomes to form intraluminal vesicles, some of which are later shed from the cell as EVs. EV content is heterogenous, derived from the parental cell, and can contain DNA, RNA, proteins, and lipids (Thery et al., 2018; Abels and Breakefield, 2016; O'Brien et al., 2020). EVs carry tetraspanins on their membrane (Thery et al., 1999; Escola et al., 1998), in particular, the tetraspanins CD81, CD9, and CD63 are highly enriched in secreted vesicles (Pegtel and Gould, 2019; Escola et al., 1998; Fordjour et al., 2022; Ai, 2024). Of these three tetraspanins, CD81 is ~15-fold more enriched in secreted small EVs, than CD63, and ~ 3-fold more than CD9, making CD81 an outstanding marker of EV biogenesis and trafficking (Fordjour et al., 2022; Ai, 2024; Mathieu et al., 2021). The recently described Exomap1 mouse takes advantage of this biology, as it is engineered for Cre-triggered expression of HsCD81 fused at its C-terminus to mNeonGreen (HsCD81mNG), allowing investigators to mark and study cell type-specific EVs *in vivo* (Fordjour et al., 2023).

Although progress has been made in understanding interacting astrocytes, there is still much to learn about their EV-mediated communication (Yu et al., 2020). Previous reports have highlighted the need for better tools to study astrocyte communication with other brain cells *in vivo* (Jurga et al., 2021; Guttenplan and Liddelow, 2019). Here, we advanced this goal by characterizing the AstroGreen mouse model that drives selective expression of HsCD81mNG in astrocytes, leading to the loading of HsCD81mNG into astrocyte-derived EVs, and allowing astrocyte-derived EVs to be detected and immunopurified using anti-human CD81 antibodies and detected by mNeonGreen fluorescence. Previous studies have shown that Cre-expressing cells can package functional Cre recombinase into EVs (Ridder et al., 2014). We leverage this finding into our AstroGreen model to demonstrate that astrocyte-derived EVs can transfer functional Cre recombinase into glioma cells *in vitro*, and into brain tumor cells *in vivo*.

## 2. Results

### 2.1. Characterization of the AstroGreen transgenic mouse model

**Fig. 1.**
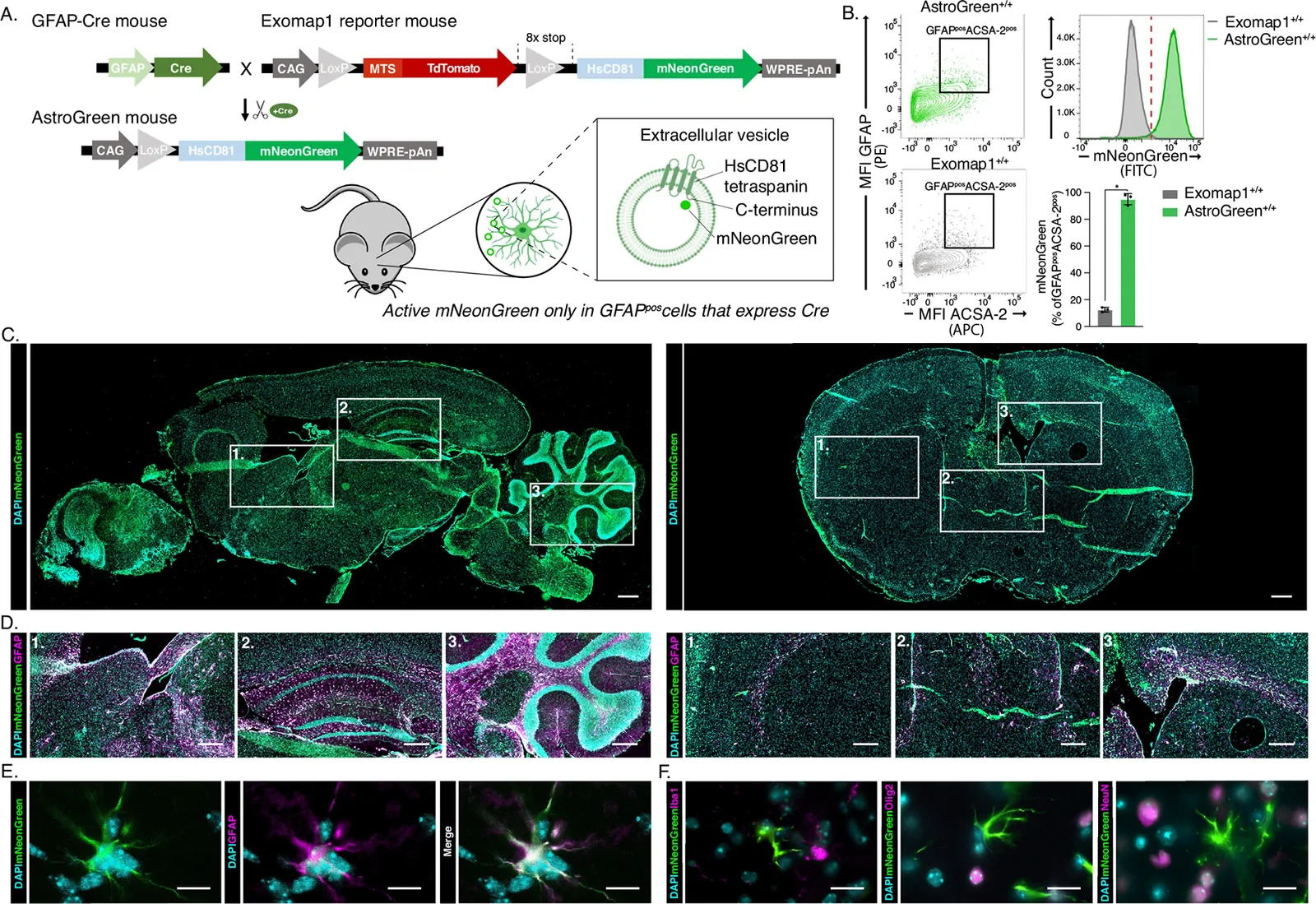
Characterization of the transgenic AstroGreen mouse model. (A) Schematic overview of how the hsCD81-GFAP^+/+^ (AstroGreen) transgenic mouse was established by crossbreeding the Exomap1 reporter mouse with the GFAP-Cre mouse model. mNeonGreen is only visible in GFAP^pos^ cells that express Cre and their secreted EVs where mNeonGreen is covalently fused to the C-terminus of the HsCD81 tetraspanin. (B) Flow cytometry analysis of AstroGreen^+/+^ – and Exomap1^+/+^-derived astrocytes gated for GFAP^POS^ACSA-2^POS^ as shown by the mean fluorescence intensity (MFI) of ACSA-2^POS^ (PE fluorophore) and GFAP^POS^ (APC fluorophore). AstroGreen astrocytes express higher levels of mNeonGreen (FITC fluorophore) as a percentage of GFAP^POS^ACSA-2^POS^ compared to Exomap1 control-derived astrocytes as quantified. Data represent three independent experiments and are presented as the mean with SEM (error bars). Multi-comparison one-way ANOVA, *p < 0.05. (C) Sagittal (left) and coronal (right) sections of the AstroGreen^+/+^ brain showing mNeonGreen (FITC) positive cells, stained for DAPI (Magnification 20×; scale bar 100 μm). (D) Zoomed-in images show co-localization of mNeonGreen (FITC) with GFAP (goat anti-rat 647) expressing cells in three locations: 1. Ventricles; 2. Hippocampus; 3. Cerebellum (Magnification 20×; scale bar 50 μm). (E) Representative images showing colocalization (merge in white) of mNeonGreen (green) and GFAP (goat anti-rat Alexa Fluor 647) of an individual astrocyte (Magnification 60×; scale bar 10 μm). (F) Other cell types in the CNS microglia (IBA1 – goat anti-rabbit Alexa Fluor 555), oligodendrocytes (OLIG2 – goat anti-rabbit Alexa Fluor 555), and neurons (NEUN – goat anti-mouse Alexa Fluor 555) (pink) were not positive for mNeonGreen (green) (Magnification 60×; scale bar 10 μm). Data represent slices of one sagittal and one coronal sectioned AstroGreen^+/+^ mouse brain.

To establish an astrocyte-specific transgenic mouse model for EV tracking, the Exomap1 transgenic mouse model (Fordjour et al., 2023) was crossed with a Cre reporter mouse under the GFAP promoter (Gregorian et al., 2009). The *exomap1* transgene, which is inserted at the Hip11 locus, carries, in order, a CAG promoter, a loxP-floxed red fluorescent protein open reading frame (ORF) (mitochondria-targeting signal (MTS)-tdTomato), the HsCD81mNG ORF. The WPRE element, was included before the polyadenylation signal to increase transgene expression and translation, by stabilizing the mRNA, leading to higher levels of the transgene product (Fordjour et al., 2023; Guo et al., 2021). The MTS-tdTomato ORF, which is expressed in most cells throughout hemizygous Exomap1^+/−^ carriers (Fordjour et al., 2023) is deleted upon Cre exposure, with MTS-tdTomato expressed in GFAP-negative cells. Membrane-localized, mNeonGreen-fluorescent HsCD81mNG is expressed in GFAP-expressing astrocytes (Fig. 1A). The homozygous AstroGreen^+/+^ mice can be reliably identified by PCR-based genotyping (Fig. S1A) using primers (Table 1) to amplify gene transcript tdTomato-HsCD81 and GFAP-Cre (Fig. S1B). Using flow cytometry, we confirmed that mNeonGreen-positive cells isolated from whole brains of homozygous AstroGreen mice were enriched for the astrocyte cell surface antigen-2 (ACSA-2) an antibody that recognizes a cell-surface epitope of the protein ATP1B, an astrocyte-specific calcium-transporting sodium-potassium ATPase, and GFAP, as compared to Exomap1 control brain (Exomap1^+/+^) (Fig. 1B). Our gating strategy showed minimal leaky expression of mNeonGreen, that we therefore consider background (Fig. S1C). In addition, we identified mNeonGreen fluorescence in sagittal and coronal brain sections (Fig. 1C) and showed that these cells also express the astrocyte marker GFAP (Fig. 1D–E). In contrast, we observed a ubiquitous expression of MTS-tdTomato in cells other than GFAP-expressing cells (Fig. S1D). We also stained neurons (NEUN), microglia (IBA1), and oligodendrocytes (OLIG2). Here we found that NEUN^pos^ cells (neurons), 1 out of a total of 257 cells co-stained for mNeonGreen (0.39 %), for OLIG2^pos^ (oligodendrocytes) 9 out of a total of 151 (5.96 %) were double positive for mNeonGreen, for IBA1^pos^ (microglia) 3 out of a total of 106 cells (2.83 %) showed colocalization of mNeonGreen, while 363 out of 367 (98.91 %) GFAP^pos^ (astrocytes) stained positive for mNeonGreen (Fig. 1F and Fig. S1E). Together, this data showed the successful generation of AstroGreen transgenic mice that express HsCD81mNG in GFAP-positive (GFAP^pos^) astrocytes.

### 2.2. Primary cultured astrocytes from AstroGreen mice express mNeonGreen

**Fig. 2.**
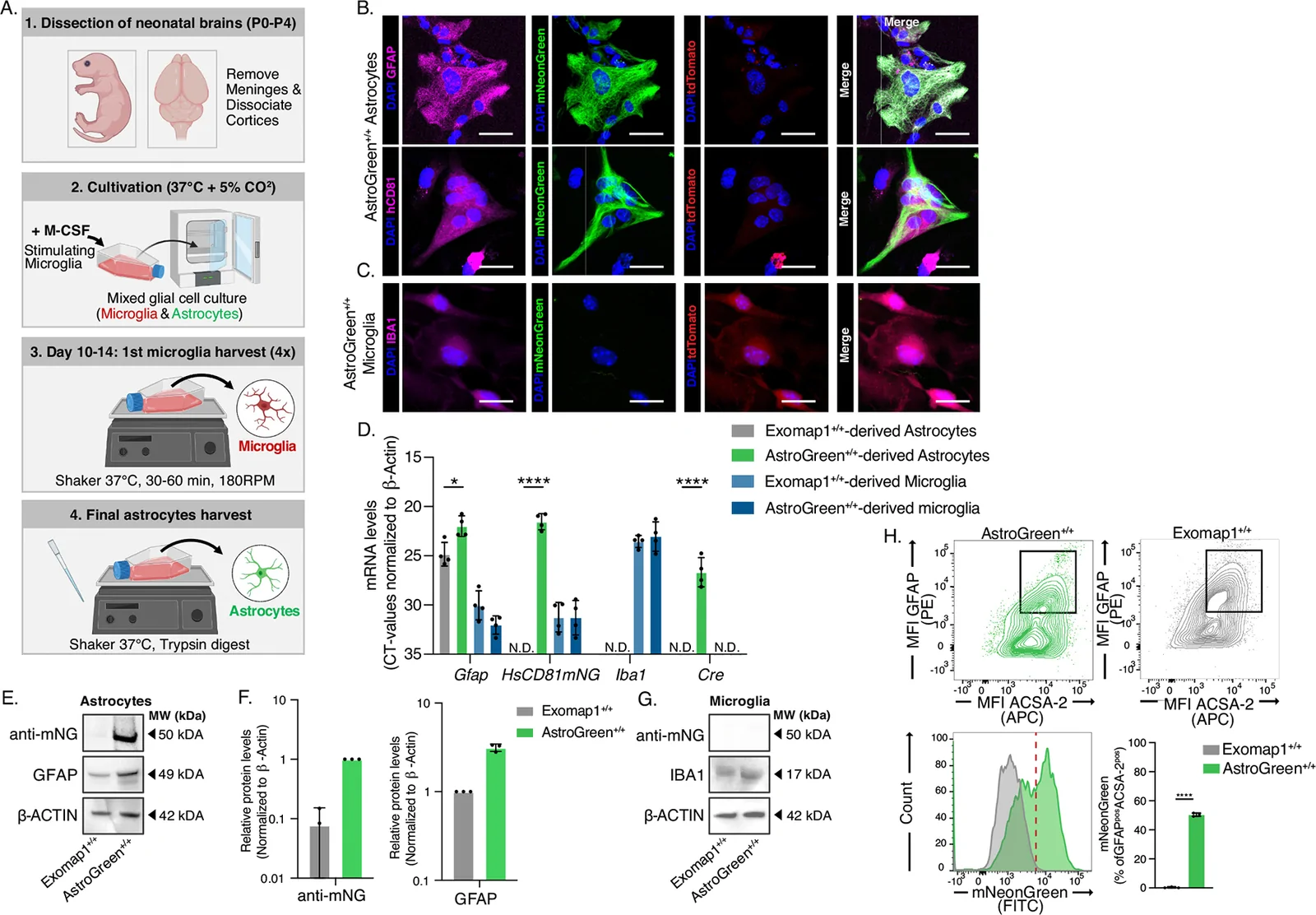
*In vitro* cultured astrocytes from AstroGreen mice express mNeonGreen. (A) Schematic overview of the method to culture primary microglia and astrocytes *in vitro*. (B) Immunohistochemistry showing co-localization of GFAP (goat anti-rat Alexa Fluor 647) and mNeonGreen (top) and overlap of HsCD81 (goat anti-rabbit Alexa Fluor 647) and mNeonGreen (bottom) in primary-derived astrocytes isolated from AstroGreen^+/+^ mixed glial cell cultures (Magnification 60×; scale bar 10 μm). (C) Immunohistochemistry of microglia derived from AstroGreen^+/+^ mixed glial cell cultures staining for IBA1 (goat anti-rat Alexa Fluor 647) were negative for mNeonGreen (Magnification 60×; scale bar 10 μm). (D) Expression levels of *Gfap*, *HsCD81mNG,* and *Cre* as determined by RT-qPCR. CT-values showed a significant increase in mNeonGreen expression in AstroGreen^+/+^ primary-derived astrocytes (green) as compared to those from Exomap1^+/+^ (grey). Microglia derived from the same mixed glial culture showed high levels of *Iba1* but low expression of HsCD81mNG for both Exomap1^+/+^ (dark blue) and AstroGreen (light blue) derived microglia, normalized to the housekeeping gene β*-Actin.* Datapoints represent four independent biological replicates and are presented as the mean with SEM (error bars). (N.D. = not determined). (E) Western blot analysis shows protein levels of GFAP, HsCD81mNG (probed against anti-NG), and β-ACTIN in primary-derived astrocytes from Exomap1^+/+^ and AstroGreen^+/+^ astrocytes. Representative images are showing a single biological sample. (F) Quantification of relative protein levels of anti-mNG and GFAP normalized to β-ACTIN as assessed on western blots. Datapoints represent three independent biological replicates. (G) Western blot analysis of microglia reveals the presence of β-ACTIN and IBA1, but not HsCD81mNG (probed against anti-NG) in primary-derived microglia from same mice as in E. Representative images are showing a single biological sample. (H) Flow cytometry analysis of AstroGreen^+/+^ – and Exomap1^+/+^ primary cultured astrocytes gated for GFAP^POS^ACSA-2^POS^ as shown by the mean fluorescence intensity (MFI) of ACSA2^POS^ (PE fluorophore) and GFAP^POS^ (APC fluorophore). AstroGreen^+/+^ astrocytes express higher levels of mNeonGreen (FITC fluorophore) as a percentage of GFAP^POS^ACSA-2^POS^ compared to Exomap1^+/+^ control-derived astrocytes as quantified. Data represent three independent experiments of three independent biological replicates per group and are presented as the mean with SEM (error bars). Multi-comparison one-way ANOVA, *p < 0.05, ****p < 0.0001.

To study the astrocytes derived from AstroGreen transgenic mice, we isolated primary neonatal astrocytes from homozygous AstroGreen^+/+^ transgenic mice, and also from Exomap1 control mice, and cultured them as previously described (Fig. 2A) (Zelenka et al., 2022). Primary astrocytes derived from AstroGreen and Exomap1 neonatal pups express the astrocyte-specific transcripts *Gfap, Aldh1a1* and *Slac1a3* (Fig. S2A). Microglia derived from the same primary mixed glial cultures expressed microglia-specific markers *Iba1, Tmem119,* and *P2ry12* (Fig. S2B). We visualized co-expression of GFAP and mNeonGreen; expectedly, mNeonGreen expressing cells were also positive for HsCD81 (Fig. 2B) Moreover, primary cultured AstroGreen astrocytes expressed ACSA-2 but did not stain for the astrocyte marker ALDH1a1, an astrocytic differentiation marker expressed in more matured astrocytes (Fig. S2C) (Adam et al., 2012). Microglia, positive for IBA1 (Fig. 2C), and TMEM119 (Fig. S2D) derived from AstroGreen^+/+^ mixed glial cultures did not express HsCD81mNG. Gene expression levels confirmed that primary-derived astrocytes from both AstroGreen^+/+^ and Exomap1^+/+^ were positive for *Gfap*, but only AstroGreen-derived primary astrocytes showed expression of *HsCD81mNG* and *Cre* mRNA (primer set sequences in Table 2). As a negative control we used primary microglia derived from the same mixed glial cultures and found that *HsCD81mNG* and *Cre* mRNAs were not detected (Fig. 2D). In the course of these experiments, we became aware that the mRNA encoded by the Exomap1 transgene was consistently less abundant in the Exomap1^+/+^ astrocytes than the AstroGreen^+/+^ astrocytes, even though its abundance was similar in microglia from these two strains. Fortunately, this unexpected feature of Exomap1 mRNA abundance in astrocytes has no bearing on the interpretations of our experiments or conclusion (Fig. 2D). The expression of HsCD81mNG in AstroGreen mice was also confirmed by immunoblot, as primary astrocytes derived from AstroGreen pups were positive for HsCD81mNG and GFAP. Interestingly, AstroGreen^+/+^ mice have ~3-fold higher levels of GFAP protein than controls (Fig. 2E). AstroGreen^+/+^ and Exomap1^+/+^ primary-derived astrocytes also expressed the astrocyte proteins AQP4, GLUT-1 and CNX43 (Fig. S2E) Immunoblots were quantified, demonstrating HsCD81mNG expression in AstroGreen^+/+^ mice, compared to Exomap1^+/+^ control (Fig. 2F). Primary-derived microglia from the same mixed glial cultures were positive for IBA1, but not for HsCD81mNG (Fig. 2G). Representative contour plots of flow cytometry data show that ~50 % of astrocytes characterized by GFAP, and ACSA-2 express mNeonGreen. AstroGreen GFAP^pos^ACSA-2^pos^ astrocytes expressed significantly higher levels of HsCD81mNG, compared to Exomap1^+/+^ control GFAP^pos^ACSA-2^pos^ astrocytes (Fig. 2H). Thus, primary-derived astrocytes, but not microglia, isolated from AstroGreen neonatal pup brains, expressed both HsCD81mNG and CRE. These data confirm the specificity of Cre expression and the subsequent recombination event leading to HsCD81mNG expression in astrocytes.

### 2.3. AstroGreen-derived extracellular vesicles contain mNeonGreen

**Fig. 3.**
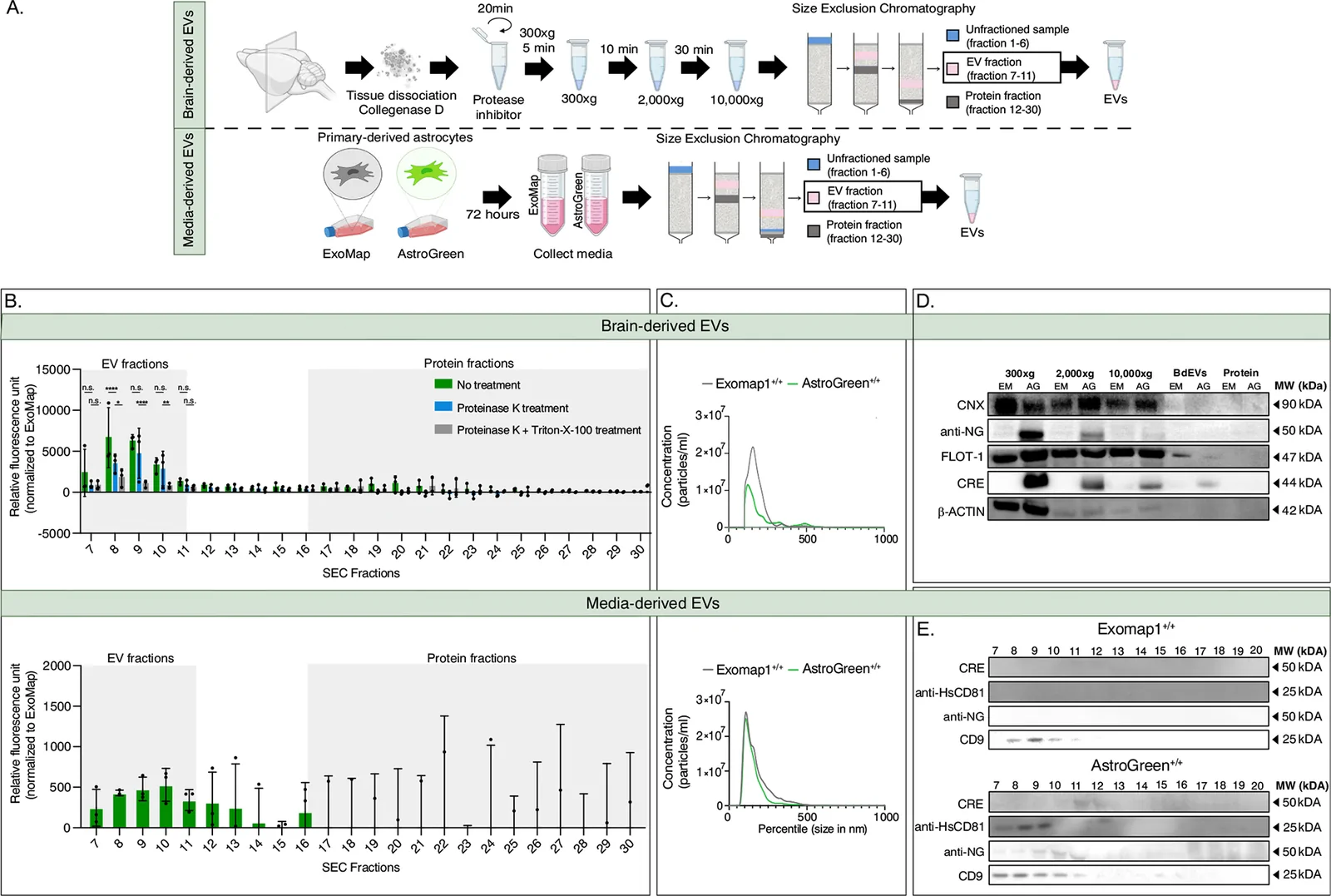
AstroGreen-derived extracellular vesicles display mNeonGreen. (A) Schematic illustration of the method used to isolate BdEVs from brains (top) and MdEVs from conditioned media of primary cultured astrocytes (bottom), EVs were collected using SEC. Fractions 7–11 were pooled and defined as EVs, and fractions 12–30 were pooled and termed protein fractions. (B) Fluorescence of mNeonGreen (Emission: 500 nm Excitation: 520 nm) of all individual fractions (7–30) from MdEVs (top) and BdEVs (bottom) were measured. Fractions were treated with proteinase K alone (blue) or with a combination of proteinase K and Triton X-100 (grey) and measured for fluorescence for mNeonGreen. Data represent three independent experiments and are presented as the mean with SEM (error bars), unpaired *t*-test *p < 0.05, **p < 0.01, ****p < 0.001, not significant (n.s.). (C) Particle count of Exomap1^+/+^ and AstroGreen^+/+^ BdEVs (top) by NTA with a total average concentration of 9.2 × 10^8^ ± 5.4 × 10^7^ (AstroGreen^+/+^) and 4.3 × 10^9^ ± 9.6 × 10^7^ (Exomap1^+/+^) particles/ml, and an average size of 120.8 ± 103.3 (AstroGreen^+/+^) and 144.7 ± 76.2 nm (Exomap1^+/+^) normalized to the weight of brain tissue. MdEVs (bottom), derived from 90 % confluent flasks incubated with media for 72 h, measured a similar particle count between Exomap1^+/+^ and AstroGreen^+/+^ by using NTA for MdEVs with total average concentration of 2.9 × 10^9^ ± 8.0 × 10^7^ (AstroGreen^+/+^) and 2.1 × 10^9^ ± 7.0 × 10^7^ (Exomap1^+/+^) particles/ml and an average size of 112.3 ± 72.9 (AstroGreen^+/+^) and 142.5 ± 73.9 nm (Exomap1^+/+^). The concentration (particles/ml) was plotted against the percentile (size in nm) AstroGreen^+/+^ and Exomap1^+/+^ for both BdEVs (top) and MdEVs (bottom) Data represent three independent experiments. (D) Western blots show anti-mNG and CRE only in AstroGreen^+/+^ BdEVs. FLOT-1 was present in all EVs, but Calnexin (CNX) was only detected in the 300 ×g, 2000 ×g, and 10,000 ×g pellets but not in BdEVs (EM = Exomap1; AG = AstroGreen). Images represent a single biological sample. (E) Protein levels of MdEVs were analyzed for single fractions (fractions 7–20 individually) and showed CD9 was present in fractions 7–11 of both AstroGreen^+/+^ and Exomap1^+/+^, however, HsCD81mNG (probed with anti-NG and anti-HsCD81 antibodies) and CRE were only present in AstroGreen^+/+^ fractions. Images represent a single biological sample.

Previous analysis of Exomap1 mice (Fordjour et al., 2023), established following Cre recombination, HsCD81mNG is delivered to the plasma membrane and loaded into EVs *in vitro* and *in vivo*, allowing the resulting EVs to be detected using human-specific anti-CD81 antibodies and mNeonGreen fluorescence. Here, we characterized the astrocyte-derived EVs from brain tissue digest solutions and primary cultured astrocytes as schematically illustrated (Fig. 3A). To isolate brain-derived EVs (BdEVs) from brain tissue of adult AstroGreen^+/+^ and Exomap1^+/+^, previously reported protocols that enzymatically dissociate brain tissue followed by size exclusion chromatography (SEC) were used (Vella et al., 2017; Rufino-Ramos et al., 2023; Huang et al., 2020). For media-derived EVs (MdEVs) conditioned cell culture media from primary-derived astrocytes cultured from newborn mouse brains were applied to SEC to isolate EVs. In both preparations, fractions 7–11 were expected to contain EV fractions, 12–30 were expected to contain free protein, and fractions 1–6 were the void volume (unfractioned) (Fig. 3A) (Monguio-Tortajada et al., 2019; Rufino-Ramos et al., 2022). We measured the fluorescence of mNeonGreen in each of the SEC fractions obtained (7–30). Both BdEVs (top) and media-derived EVs (MdEVs - bottom) derived from AstroGreen^+/+^ mouse brains and cultured astrocytes, respectively, showed increased expression of mNeonGreen fluorescence in fractions 7–11 normalized to Exomap1^+/+^ control-derived EVs from the same sources (Fig. 3B). Notably, BdEVs show ~30-fold higher levels of mNeonGreen fluorescence compared to MdEVs. However, after each fraction (7–30) was incubated with proteinase K, mNeonGreen fluorescence in BdEVs protein fractions (12–30) was lowered after proteinase K treatment, while those in EV fractions (7–11) were not, indicating that mNeonGreen is associated and protected by the membrane of the EVs. In contrast, EVs that we incubated with both proteinase K and Triton-X-100 showed a disrupted membrane due to the Triton-X-100 treatment allowing Proteinase K to degrade mNeonGreen as observed by lower mNeonGreen fluorescence. Consequently, the mNeonGreen signal dropped, indicating that mNeonGreen present in the lumen of the EVs was digested by Proteinase K upon membrane disruption (Fig. 3B). Next, fractions 7–11 were pooled from either the brain or cultured astrocytes and quantified by Nanoparticle Tracking Analysis (NTA). Particle counts of Exomap1^+/+^ and AstroGreen^+/+^ BdEVs were a total average concentration of 9.2 × 10^8^ ± 5.4 × 10^7^ (AstroGreen^+/+^) and 4.3 × 10^9^ ± 9.6 × 10^7^ (Exomap1^+/+^) particles/ml ^+/+^, normalized to the weight of brain tissue used, with an average size of 120.8 ± 103.2 (AstroGreen^+/+^) and 144.65 ± 76.2 nm (Exomap1^+/+^). For MdEVs we measured a similar particle count between Exomap1^+/+^ and AstroGreen^+/+^ by using NTA with total average concentration of 2.9 × 10^9^ ± 8.0 × 10^7^ (AstroGreen^+/+^) and 2.2 × 10^9^ ± 7 × 10^7^ (Exomap1^+/+^) particles/ml with an average size of 112.3 ± 72.9 (AstroGreen^+/+^) and 142.5 ± 73.9 nm (Exomap1^+/+^). The concentration (particles/ml) was plotted against the size (in nm) for AstroGreen^+/+^ (green) and Exomap1^+/+^ (grey) mice for both BdEVs (top) and MdEVs (bottom) (Fig. 3C). No differences between EV size were observed for both BdEVs and MdEVs as measured by NTA (Fig. S3A). Two primer sets (HsCD81mNG-1 and HsCD81mNG-2; see Table 2) flanking two different locations in the HsCD81mNG transgene were used to measure mRNA levels normalized to particle number. Here, we found that the levels of Cre mRNAs were 200-fold higher in BdEVs compared to MdEVs (Fig. S3B), indicating that BdEVs carry more Cre mRNA than MdEVs, however this could also be due to fragmentation of cells into EV-like structures. HsCD81mNG mRNA levels did not differ between Exomap1^+/+^ and AstroGreen^+/+^ BdEVs. In Exomap1^+/+^ BdEVs, the levels of *tdTomato* mRNA were 3-fold higher and *Iba1* levels were increased 5-fold compared to AstroGreen^+/+^ BdEVs. In Exomap1^+/+^-derived MdEVs *tdTomato* was expressed with a 4-fold difference compared to AstroGreen^+/+^-derived MdEVs. *Iba1* expression was low in both AstroGreen^+/+^ and Exomap1^+/+^-derived MdEVs, this is expected because the MdEVs were isolated from media that was incubated with primary astrocytes cultures that do not contain microglia (Fig. S3C). Western blots confirmed that HsCD81mNG and CRE were only present in BdEVs isolated from AstroGreen^+/+^ (AG) mice, and not in BdEVs from Exomap1^+/+^ (EM) mice (Fig. 3D and Fig. S6). Flotillin-1 (FLOT-1), a protein marker typically associated with EVs, was enriched in all EV fractions, but calnexin (CNX), an endoplasmic reticulum membrane protein, was present in the 300 ×g, 2000 ×g and 10,000 ×g pellets, collected after each centrifugation step, but was undetectable in the small BdEVs that pellet at 100,000 ×g (Fig. 3D). Protein expression in MdEVs fractions showed the presence of HsCD81mNG and CRE in fraction 7–12, but not in fractions 13–30 in AstroGreen-derived EVs compared to Exomap1^+/+^ control EVs, and mouse CD9 was found in fractions 7–11 of both AstroGreen^+/+^ and Exomap1^+/+^ (Fig. 3E and Fig. S6). Taken together, we showed that EVs isolated from AstroGreen^+/+^ mice obtained from the brain and from the media of primary cultured astrocytes derived from AstroGreen^+/+^ neonatal pups contained HsCD81mNG and Cre, whereas neither were present in parallel Exomap1^+/+^ control samples.

### 2.4. Optimizing the detection of AstroGreen features in EVs with anti-HsCD81-coated microbeads

**Fig. 4.**
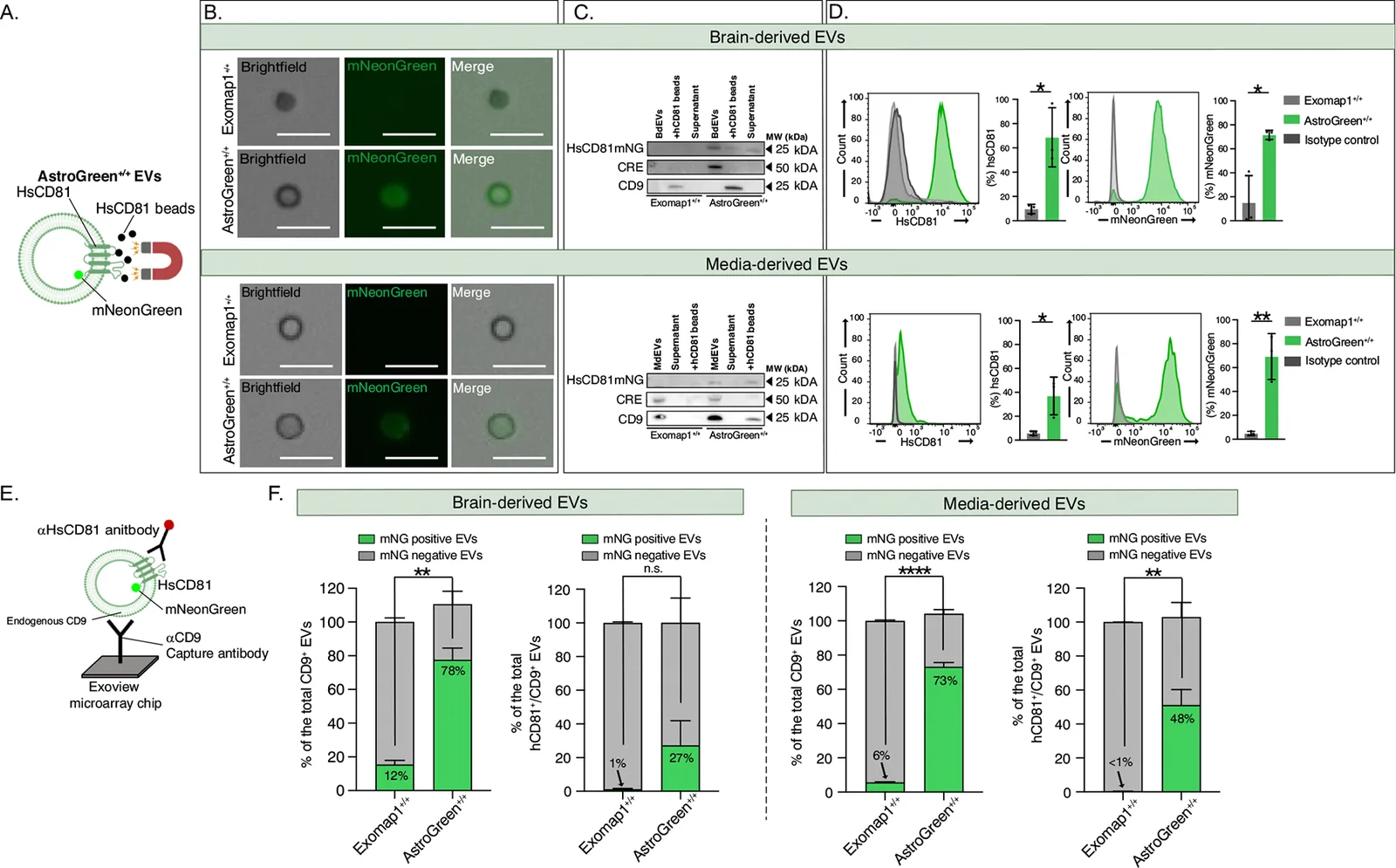
HsCD81 enriched extracellular vesicles contain HsCD81 and CRE. (A) Schematic display of an AstroGreen EV harboring mNeonGreen on the C-terminus of the HsCD81 tetraspanin bound to HsCD81 magnetic beads. (B) Representative images show AstroGreen BdEVs (top) and MdEVs (bottom) bound to HsCD81 microbeads expressing mNeonGreen (FITC) which was visualized by microscopic imaging (60× magnification, scale bar 10 μm). Images represent a single biological sample. (C) Western blot of EVs alone, EVs bound to HsCD81 beads, and the supernatant show the presence of HsCD81mNG (as probed for anti-HsCD81) and CRE only in AstroGreen, and CD9 in both AstroGreen^+/+^ and Exomap1^+/+^ for BdEVs and MdEVs. Images represent a single biological sample. (D) Flow cytometry analysis showed expression of HsCD81 (PE fluorophore) and mNeonGreen (FITC fluorophore) in BdEVs and MdEVs from AstroGreen^+/+^ but not in Exomap1^+/+^ derived EVs. Data represent three independent experiments of three independent biological replicates per group and are presented as the mean with SEM (error bars). Multi-comparison one-way ANOVA, * < 0.05, **p < 0.01. (E) Schematic representation illustrating the ExoView method we used to capture single EVs on the anti-CD9 capturing antibody and characterization of mNeonGreen and HsCD81. (F) Quantification shows the significantly higher fluorescence intensity of mNeonGreen (mNG) positive EVs as a percentage of the total CD9^+^ EVs for both BdEVs (left) (AstroGreen^+/+^ 78 %; Exomap1^+/+^ 12 %) and MdEVs (right) (AstroGreen^+/+^ 73 %; Exomap1^+/+^ 6 %). The percentage of mNG positive EVs as a total of HsCD81+/CD9+ EVs shows no significant difference in mNG in AstroGreen^+/+^ for BdEVs (AstroGreen^+/+^ 27 %; Exomap1^+/+^ 1 %) however, the percentage of mNG was significantly higher for MdEVs (AstroGreen^+/+^ 48 %; Exomap1^+/+^ < 1 %). Data represent two or three independent experiments and are presented as the mean with SEM (error bars). Multi-comparison one-way ANOVA, **p < 0.01, ****p < 0.0001, n.s. = not significant.

We next used magnetic microbeads conjugated with anti-HsCD81 antibodies to collect HsCD81-positive BdEVs and MdEVs onto the bead surface (Fig. 4A). Both HsCD81-positive BdEVs and MdEVs were pulled down selectively using HsCD81 microbeads (Fig. 4B). Protein blotting showed the presence of HsCD81mNG in AstroGreen-derived BdEVs and MdEVs bound to anti-HsCD81 beads, but not in BdEVs or MdEVs derived from Exomap1^+/+^ controls. BdEVs and MdEVs from both AstroGreen^+/+^ and Exomap1^+/+^ contained CD9. However, unpurified BdEVs lacked detectable levels of CD9 and Cre, whereas MdEVs contained both (Fig. 4C and Fig. S6). Taken together, these results confirm that HsCD81mNG-positive EVs of outside-out topology can be immunopurified on anti-HsCD81 immunocapture beads, that EVs of correct topology were enriched in CD9, HsCD81mNG, and CRE, and that BdEVs include a substantial number of HsCD81mNG-positive vesicles that are refractory to immunopurification on anti-HsCD81 magnetic beads. Next, we analyzed the anti-HsCD81 beads carrying bound BdEVs and MdEVs by staining them with a PE-labeled anti-HsCD81 antibody and then examining them by flow cytometry to analyze the amount of exposed HsCD81 antigen on the EV surface, and their amount of mNeonGreen fluorescence. Here we found differences between BdEVs and MdEVs, showing that the beads with EVs captured from the brain-suspension (BdEV) ~100-fold higher staining intensity for surface HsCD81 but ~2–3 times less mNeonGreen mean fluorescence compared to the beads with EVs captured from culture media (MdEV). This could be due to mNeonGreen getting cleaved from the HsCD81 and not getting loaded or because mNeonGreen is released as free protein (Fig. 4D). Finally, AstroGreen BdEVs and MdEVs were imaged and analyzed by the combined techniques of single-particle interferometric reflectance (SP-IR) imaging coupled to immunofluorescence microscopy using the ExoView^®^ camera a platform for single EV analysis (Fordjour et al., 2022; Breitwieser et al., 2022; Daaboul et al., 2016). Both BdEVs and MdEVs were captured by binding the endogenous CD9 tetraspanin with the mouse anti-CD9 capture antibody present on the ExoView^®^Chip. The capturing of a single EV by an antibody allows for multiparametric comparison by colocalizing fluorescent signals of endogenous mNeonGreen based on its green fluorescence and anti-HsCD81 by the red fluorophore conjugated to the detection antibody, as schematically illustrated (Fig. 4E). We analyzed the percentage of mNeonGreen-positive EVs comparing EVs derived from the AstroGreen^+/+^ or the Exomap1^+/+^ control. The mNeonGreen positive EVs as a percentage of the total CD9^+^ captured EVs was 66 % higher in AstroGreen^+/+^ in BdEVs (left) and 67 % in MdEVs (right) compared to the Exomap1^+/+^ control. The fluorescence intensity of mNeonGreen-positive EVs as a percentage of the HsCD81^+^/CD9^+^ EVs showed 27 % higher levels in AstroGreen^+/+^ BdEVs (left) and 47 % higher in AstroGreen^+/+^ MdEVs (right). Due to the auto-fluorescent nature of the brain, a large number of Exomap1 BdEVs showed positive in the fluorescent measurements (Fig. 4F). In sum, the evidence shows that mNeonGreen-positive BdEVs and MdEVs derived from the AstroGreen^+/+^ transgenic mouse model can be captured using HsCD81 antibody. In addition, the fluorescence can be monitored after capture using EV-specific antibodies.

### 2.5. Astrocyte-derived extracellular vesicles isolated from specific brain regions

Astrocytes are found ubiquitously throughout the CNS and were previously viewed as a homogenous cell type; however, over the last few years, questions have been raised as to whether distinct subtypes of specialized astrocytes exist (Khakh and Deneen, 2019). Unique subtypes of region-specific astrocytes have been identified in areas such as cortical layers, hippocampus, cerebellum, and brainstem, with various levels of GFAP expression (Jurga et al., 2021; Batiuk et al., 2020; Chai et al., 2017). To characterize astrocyte-derived EVs isolated from these different areas in the AstroGreen^+/+^ and Exomap1^+/+^ mouse brains, they were anatomically dissected and analyzed (Fig. S4A). *Gfap* levels were equal between AstroGreen^+/+^ and Exomap1^+/+^, meaning that all regions contained GFAP-expressing astrocytes (Fig. S4B). Brain tissue from the specific areas was dissociated, and 24 fractions were isolated by SEC for each, as described above. Fractions 7–30 were measured for mNeonGreen fluorescence for each specific brain region. In all structures, mNeonGreen was distinctly present in AstroGreen^+/+^ BdEVs (fraction 7–11) compared to the same fractions from the Exomap1^+/+^ control (Fig. S4C). Next, HsCD81mNG-positive EVs were enriched using anti-HsCD81 microbeads. AstroGreen-derived BdEVs showed high levels of mRNA for *HsCD81mNG* and *Cre* in all brain regions at gene expression (Fig. S4D). After normalizing the number of EVs per 10^9^ particle counts between brain regions, pooled fractions were again measured for mNeonGreen to compare fluorescence intensity among different brain structures. mNeonGreen was expressed throughout all brain areas, with the highest expression in the hippocampus, thalamus, and olfactory bulbs, the brain regions with the highest GFAP expression (Fig. S4E). BdEV fractions 7–11 of each brain area were pooled and quantified by NTA (Fig. S4F and Table 3). Additionally, plasma derived from AstroGreen^+/+^ mice had increased mNeonGreen fluorescence intensity compared to Exomap1^+/+^ control (Fig. S4G). The data presented show that the AstroGreen mouse model allows us to study astrocytes from different brain regions and provides the opportunity to study EVs isolated from specific brain areas and other tissue/fluids.

### 2.6. Functional AstroGreen-derived extracellular vesicle transfer into recipient glioma cells in vitro and in vivo

**Fig. 5.**
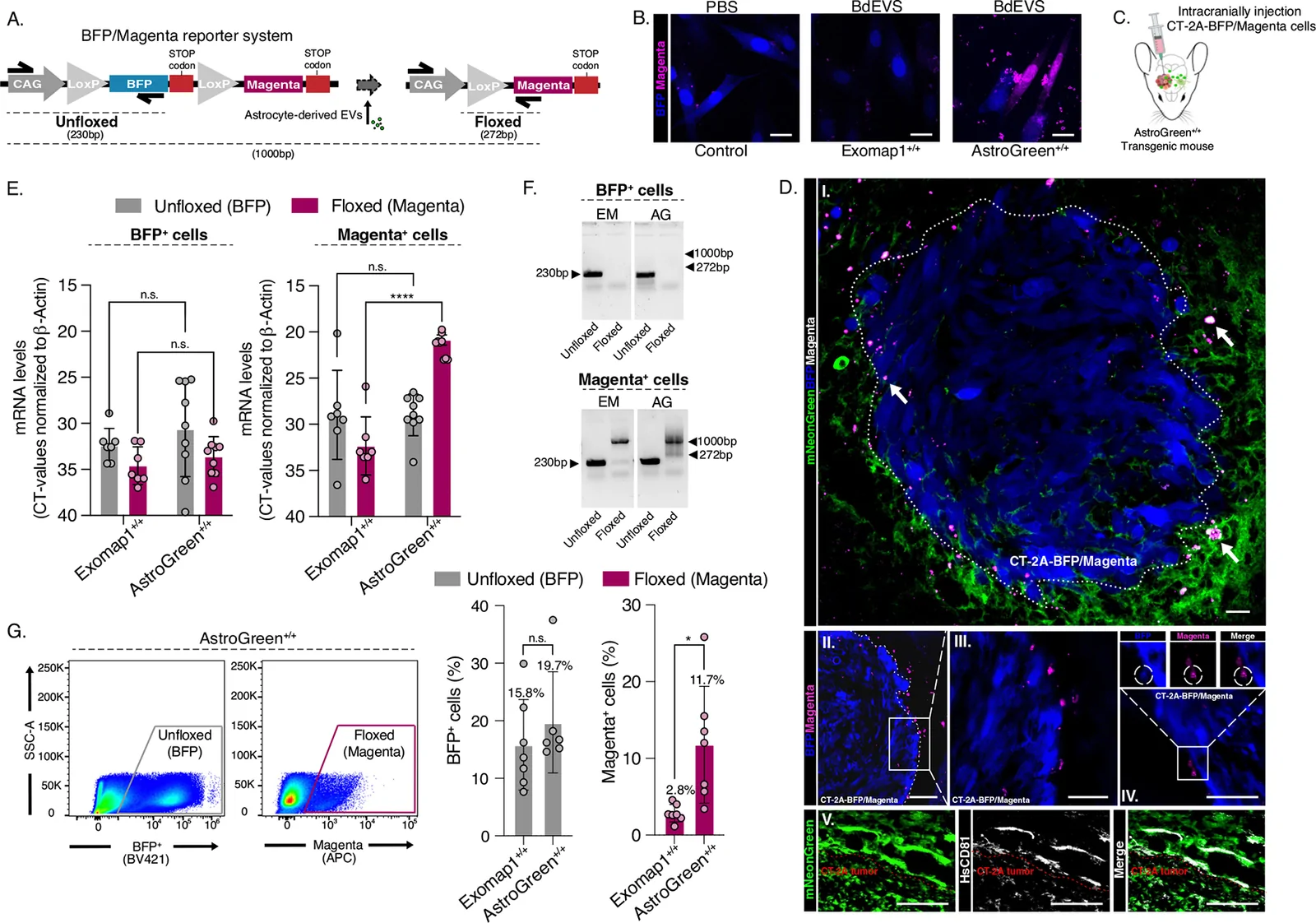
Functional transfer of Cre from AstroGreen expressing cells into recipient glioma cells *in vivo*. (A) Schematic illustration of the BFP/iRFP reporter system showing blue fluorescent protein (BFP) driven by the CAG promoter is followed by a STOP codon (STOP) flanked by two LoxP sites. Cre-driven recombination results in the deletion of the BFP followed by expression of the near-infrared fluorescent protein (iRFP) followed by a STOP codon. Primers specifically designed to amplify the floxed allele (272 bp) the unfloxed allele (230 bp) and a 1000 bp band when floxed cells still contain the unfloxed allele. (B) Representative images of three biological replicates of CT-2A-BFP(blue)/iRFP(magenta) cells showing iRFP^+^ floxed cells post-transfection incubated for 5-days with AstroGreen^+/+^ or Exomap1^+/+^ BdEVs compared to (PBS) the negative control. BdEVs were resuspended in PBS and concentrations were measured by NTA. Cultured cells (1 × 10^5^) were exposed with a single dose of (2 × 10^9^ particles) and incubated for 5-days. (Scale bar 10 μm) (C) Schematic display of an AstroGreen^+/+^ transgenic mouse intracranially injected in the left striatum with CT-2A mouse glioma cells stably expressing BFP/iRFP reporter. (D) Representative images of three biological samples of CT-2A-BFP unfloxed tumor cells (blue) and floxed cells (iRFP (in magenta) at the tumor border (white dotted line) 14 days post-implantation in the left striatum of an AstroGreen^+/+^ mouse. mNeonGreen positive astrocytes show HsCD81 (white) at the tumor border and inside the tumor. Tumor border highlighted with a red dashed line. AstroGreen^+/+^ astrocytes (green) (I. magnification 20×; scale bar 10 μm; white arrows point at floxed cells. II. magnification 4×; scale bar 10 μm; III. magnification 20 × 10 μm; IV. magnification 40×; scale bar 10 μm; V. magnification 40×; scale bar 10 μm). (E) Representative images of two CT-2A tumor-bearing AstroGreen+/+ brains at day 14 post-tumor implantation with mNeonGreen positive astrocytes shows HsCD81 (white) at the tumor border and inside the tumor. Tumor border highlighted with a red dashed line. (Magnification 40×; scale bar 50 μm). (F) After FACS sorting of BFP^+^ and iRFP^+^ tumor cell populations, the unfloxed and floxed allele expression levels were analyzed with RT-qPCR. The AstroGreen^+/+^ iRFP sorted cells showed significantly increased expression levels of the floxed allele compared to the Exomap1^+/+^ control. No significant difference was observed between the groups in the BFP^+^ population. Data represent three independent experiments (total mice *n* = 9) and are presented as the mean with SEM (error bars). Multi-comparison one-way ANOVA, **** < 0.0001, n.s. = not significant. (G) The 272 bp amplicon of the floxed allele was only determined in the iRFP^+^ sorted population and not in BFP sorted cells. The unfloxed allele (230 bp) band was only present in BFP-sorted cells. The 1000 bp observed band represents unfloxed cells in the floxed group meaning that not all iRFP^+^ cells were completely floxed. Images represent three technical and biological samples. (H) Representative flow cytometry plots show gating of the BFP (grey box) and iRFP (reddish purple box) populations. Quantification showed the percentage of BFP^+^ (15.8 % Exomap1^+/+^ and 19.7 % AstroGreen^+/+^) and iRFP^+^ events comparing AstroGreen^+/+^ and Exomap1^+/+^ tumor-bearing mice. AstroGreen^+/+^ mice had four times as many iRFP^+^ cells (11.7 %) compared to Exomap1^+/+^ mice (2.8 %). Data represent three independent experiments (total mice *n* = 7) and are presented as the mean with SEM (error bars). Multi-comparison one-way ANOVA, * p < 0.05.

Previous studies have shown that astrocytes shed EVs and that astrocyte-derived EVs may impact neuroprotection (Pascua-Maestro et al., 2018), neuronal function (You et al., 2020), and disease pathology (Gharbi et al., 2020; Zhao et al., 2021; Upadhya et al., 2020). To determine whether astrocyte-derived Cre mRNA and protein might be transferred to glioma cells, we used a Cre reporter that encodes BFP flanked by two lox sites, followed by a stop codon, and a near-infrared fluorescent protein (iRFP731) (Obuchi et al., 2024) (Fig. 5A). Primer sets were specifically designed to distinguish between the unfloxed allele (amplicon 230 bp) and the floxed allele (amplicon 272 bp), a 1000 bp band is observed when cells are double positive for BFP and iRFP, indicating that floxed cells still contain the unfloxed allele (Table 2). After we stably transduced the CT-2A mouse glioma cell line with the BFP-iRFP reporter transgene in culture, we analyzed Cre activity by the detection of iRFP^+^ (in magenta) CT-2A cells *in vitro* following a 5-day exposure to PBS (negative control), or a single dose of (2 × 10^9^ particles) Exomap1^+/+^-derived BdEVs, or AstroGreen^+/+^-derived BdEVs. Floxed events were observed when CT-2A-BFP/iRFP cells were incubated with AstroGreen^+/+^-derived BdEVs (Fig. 5B). Control experiments established that the addition of AstroGreen^+/+^-derived MdEVs induced the Cre-dependent appearance of iRFP^+^ cells whereas MdEVs from Exomap1^+/+^ cells did not (Fig. S5A–B) (Madisen et al., 2010). We also showed that exposure of these reporter cells to AstroGreen^+/+^ BdEVs or MdEVs triggered the appearance of the 272 bp-long PCR product that is diagnostic for the floxed allele, but not in cells exposed to Exomap1^+/+^-derived EVs (Fig. S5B). The amplification of the floxed allele (272 bp) was only observed in CT-2A-BFP/iRFPcells transfected with a Cre-expressing construct or exposed to AstroGreen^+/+^ BdEVs or MdEVs (Fig. S5C). CT-2A-BFP/iRFP cells were injected intracranially into the left striatum of the AstroGreen^+/+^ (Fig. 5C) or Exomap1^+/+^ mice (Fig. S5D). Mice were sacrificed 14 days after tumor implantation, both iRFP^+^ cells and HsCD81^+^ cells were detected at the border of the CT2A-BFP/iRFP tumor only in AstroGreen^+/+^ mice (Fig. 5D–E). Both BFP^+^ and iRFP^+^ tumor cell populations were FACS sorted, no significant difference was observed between the groups in the BFP^+^ population in mRNA expression of the unfloxed and floxed transcript. The AstroGreen^+/+^ iRFP sorted cells showed significantly increased expression levels of the floxed allele compared to the Exomap1^+/+^ control (Fig. 5F). In addition, the 272 bp amplicon of the floxed allele was only determined in the iRFP^+^ sorted population (Fig. 5G). Flow cytometric analysis showed the percentage of BFP^+^ and iRFP^+^ events comparing AstroGreen^+/+^ and Exomap1^+/+^ tumor-bearing mice. Gating strategy of CT-2A and CT-2A-BFP/iRFPcells to determine BFP^+^ and iRFP^+^ cell populations (Fig. S5E). AstroGreen^+/+^ mice implanted with the CT-2A-BFP/iRFP tumor cells had 2 times more iRFP^+^ cells detected compared to Exomap1^+/+^ (Fig. 5H). To determine the implication of the HsCD81 or CRE expression on the overall health of the animal's pathology analysis were performed. Here it was found that both transgenic mouse models (AstroGreen^+/+^ and Exomap1^+/+^) showed that liver markers were within normal range, suggesting that HsCD81 expression does not affect the health of the mice (Fig. S7A). Overall, we have shown a direct transfer of functional CRE molecules from astrocytes to glioma cells mediated by AstroGreen EVs and potentially other extracellular mechanisms.

## 3. Discussion

This study characterized a transgenic mouse model (AstroGreen) that can be used to monitor the release and uptake of astrocyte-derived EVs. This transgenic mouse model allows tracking of HsCD81-positive/fluorescently labeled EVs released by GFAP-expressing astrocytes in culture and *in vivo*. We validated that only GFAP-positive cells in the AstroGreen^+/+^ brains express mNeonGreen fluorescence, both early developmental stage (day 0–4) and in adult mice (week 10–15). In addition, only astrocytes isolated from primary-derived mixed glial cultures dissociated from neonatal pups expressed mNeonGreen fluorescence, but not the microglia derived from the same pups indicating that mNeonGreen fluorescence in these animals was specific to astrocytes. We found that both BdEVs and MdEVs carried HsCD81mNG protein, and HsCD81mNG mRNA inside their EVs. Additionally, Cre mRNA and protein were also found in both BdEVs and MdEVs. Notably, BdEVs showed 10 times higher HsCD81 mRNA and 200 times more Cre mRNA compared to MdEVs. A similar pattern was found in different areas of the brain, where EVs isolated from different brain regions contained HsCD81mNG and Cre species, serving as important reporter markers to study brain region-specific astrocytes and their EVs. Finally, using this mouse model we could observe functional delivery of CRE from astrocytes to tumor cells. A CT-2A-BFP/iRFP reporter cell model that triggers iRFP expression upon Cre recombination allowed us to visualize and quantify direct cargo transfer from astrocytes to glioma cells in culture and *in vivo*.

Here we demonstrated that the HsCD81mNG transgene is expressed in GFAP-positive astrocytes and that astrocyte-derived HsCD81-positive EVs can transfer functional Cre into recipient cells *in vitro.* We also observed that intracranial implantation of glioma cells into the striatum of AstroGreen mice resulted in the detection of mNeonGreen fluorescence and HsCD81 in the tumor mass, and in the functional uptake of astrocyte-derived Cre species by glioma cells, especially those at the periphery of the tumor mass and in the glioma cells that had migrated into the surrounding stroma. However, we did not see astrocyte-mediated transfer of mNeonGreen fluorescence towards other cell types in the CNS. This might be explained by the finding that brain EV communication is a time-dependent process that requires long-term exposure to deliver enough EVs to cells and mediate a measurable and visible effect (Rufino-Ramos et al., 2023). Other brain regions showed no presence of mNeonGreen, which could be due to the limitation that mNeonGreen transferred by EVs is not easily detected in the brain tissue which has widespread green autofluorescence. However, this could be solved by HsCD81 immunostaining, which affords a greater fluorescence intensity and specificity than mNeonGreen fluorescence alone. Additionally, we observed mNeonGreen signal in the plasma from AstroGreen mice, suggesting GFAP+ EVs being secreted into the blood stream. Circulating EVs can cross the blood brain barrier (BBB) in a bidirectional manner, however the routes of transport between the bloodstream and brain parenchyma remain poorly understood (Ramos-Zaldivar et al., 2022). Active transcytosis has been suggested as the most likely pathway that EVs use to cross of the healthy BBB (Matsumoto et al., 2017). In a diseased condition, the BBB is more permeable and leakier which makes it easier to track EVs that crossed a disrupted BBB (Saint-Pol et al., 2020). EVs can be directly secreted to the brain fluids such as CSF and blood and it has been evaluated that EVs could serve as potential biomarkers to improve diagnostics and follow-up in high-grade glioma (Ricklefs et al., 2024).

In the past, brain-derived EVs, encompassing “exosomes”, have been isolated after papain digestion and homogenization or filtration which are mechanical methods that typically disrupt tissues (Perez-Gonzalez et al., 2012; Gallart-Palau et al., 2016). We acknowledge that the interstitial space between tissue can potentially compromise the extracellular environment and fragmentation of cell membranes could contaminate the isolated BdEVs. The membranous material isolated by these methods have previously been called “exosomes”, although the experimental requirements set out by the International Society for Extracellular Vesicles (Thery et al., 2018; Welsh et al., 2024; Lotvall et al., 2014; Hill et al., 2013) are not totally in accordance. In this study, we used a recently optimized method to enrich EVs populations from brain tissue, previously described by Vella et al., 2017. In this protocol EVs were extracted from tissue after collagenase treatment and less mechanical action. Then, tissue-derived EVs were concentrated and isolated by SEC. EVs were characterized based on their morphology, size, and density, and their cargo was studied on a proteomic and RNA level (Vella et al., 2017; Huang et al., 2020). So far, this BdEV isolation method has been used by several groups (Rufino-Ramos et al., 2023; Huang et al., 2022; Breyne et al., 2022; Su et al., 2021; D'Acunzo et al., 2022). In addition, recent efforts have been made to provide a human BdEV atlas of EVs derived from different regions of the brain (Huang et al., 2023). Despite enrichment of BdEVs based on traditional EV markers, including CD9 and FLOT-1, and undetectable levels of the CNX marker, further steps could be taken to eliminate the co-isolation of, for example, DNA originating from nuclei disruption, protein contaminants in BdEV preparations and non-EV particles that overlap EVs in size and morphology. We therefore recognize the possibility that our BdEVs samples could contain some non-EV particles generated by our protocol which might account for the observed substantial differences between BdEVs and MdEVs. Specifically, we found that BdEVs were distinct from MdEVs in that their mNeonGreen fluorescence was ~30-fold higher, their levels of mRNA were 10-fold higher for HsCD81mNG and 200-fold higher for Cre, their immunostaining with anti-HsCD81mNG antibodies was ~100-fold higher, and ~ 50 % of BdEVs could not be captured on anti-HsCD81 antibody beads, likely because of inefficient pulldown rather than intrinsic biological properties. Future studies are needed to elucidate the molecular mechanisms that underly these substantial differences between tissue-derived and cell-derived EVs and raise the possibility that there may be limitations to tissue-derived EV isolation methods (Brenna et al., 2021; Crescitelli et al., 2021). Notably, primary cultured astrocytes isolated from neonatal pups are selectively derived from the cortices which results in a more homogenous MdEV population. In contrast the BdEVs are derived from slices throughout the entire adult brain, containing multiple brain regions resulting in a more mixed BdEV sample, which in part might account for the observed differences between BdEVs and MdEVs. In addition, to control for the changes in composition and function of the EVs when using genetically engineered models' comparison to wild-type control is crucial to ensure that these genetical changes are not resulting in aberrant EV function.

Previous studies have shown that healthy glia secrete EVs that interact with neighboring cells, modulating neuronal differentiation and activation (You et al., 2020; Fruhbeis et al., 2013), promoting microglia activation (Paolicelli et al., 2019; Liao et al., 2020) and regulating angiogenesis and BBB function (Pivoriunas and Verkhratsky, 2021). Upon ATP stimulation, astrocytes release an increased number of EVs (Drago et al., 2017), with altered EV cargo, including an increase in proteins such as - dihydropyrimidinase like 2 (DPYSL2) and secreted protein acidic and cysteine-rich (SPARC), both of which are involved in regulating neuronal excitability (Chaudhuri et al., 2018; Datta Chaudhuri et al., 2020). Communication through astrocyte-derived EVs has been implicated in both promoting and suppressing the progression of various brain diseases, such as Alzheimer's disease, Parkinson's disease, stroke, glioma, and aging (Zhao et al., 2021; Upadhya et al., 2020; Pei et al., 2019; Rouillard et al., 2021; Broekman et al., 2018; Edwardson et al., 2024). It has been suggested that astrocyte-mediated EV communication in glioma pathology has a tumor-supportive role (Nieland et al., 2021). Although efforts have been made to study EV communication between astrocytes and glioma (Zeng et al., 2020; Serpe et al., 2022; Koessinger et al., 2023), the majority of studies were based on co-culture approaches (Nieland et al., 2021; Oushy et al., 2018; Hallal et al., 2019; Colangelo and Azzam, 2020) and these effects have not been conclusively established *in vivo* (Patel and Weaver, 2021; Mahjoum et al., 2021). Therefore, the establishment of models and improved techniques that allow the investigation of astrocyte-derived EV communication offers a new perspective in the field (Yu et al., 2020).

Despite sophisticated animal models developed to study neuronal-derived EV uptake by astrocytes (Gao et al., 2020; Men et al., 2019), it is important to note that astrocytes interact not only with neurons but with a variety of cell types (*i.e.*, microglia, fibroblasts, pericytes) in their surroundings. Advanced transgenic animal models have been reported including a transgenic mouse model expressing the tetraspanin CD9 fused to EGFP (EVRep) (Norgard et al., 2022), and the transgenic inducible GFP EV reporter (TIGER) mouse model expressing inducible CD9-GFP in a cell-type specific manner. CD9-GFP labeled cells included GFAP-positive astrocytes, allowing the study of astrocyte-derived CD9-positive EVs (Neckles et al., 2019). Other transgenic models described previously are based on tetraspanin CD63 expression labeled with a fluorescent protein (Men et al., 2019; Yoshimura et al., 2016; Yoshimura et al., 2018; McCann et al., 2020; Li et al., 2022). However, CD63 protein is less enriched in EVs compared to CD9 and CD81 (Fordjour et al., 2022), and high levels of CD63 might affect EV content (Ai et al., 2023). Notably, CD63 is reported lethal when overexpressed in rats (Yoshimura et al., 2016) and associated with contamination with larger, round, non-EV particles (Zuppone et al., 2023), altogether making it unclear if CD63 is an appropriate EV-related marker to study EV biogenesis.

Fusing proteins to the C-terminus of tetraspanins like CD81 can influence their trafficking and incorporation into EVs, with effects highly dependent fusion design. For instance, adding endocytosis motifs to the C-terminus of CD81 disrupts its EV incorporation and leads to protein degradation (Ai et al., 2023). In contrast, engineered fusions to CD81, such as GFP have been successfully used to load proteins into EVs without impairing their secretion or natural trafficking (Silva et al., 2021; Gurrieri et al., 2024). Similarly, CD9, a close tetraspanin relative of CD81, shows comparable EV behavior and structural sensitivity. Recent live-cell imaging studies revealed that CD9 and CD63 initially traffic together through the ER and Golgi, but later diverge. CD9 localizes primarily to the plasma membrane, while CD63 accumulates in acidic compartments like late endosomes and lysosomes (Mathieu et al., 2021). This divergence highlights distinct trafficking routes that may influence how C-terminal tags affect their function and localization. Interestingly, although CD9, CD81, and CD63 are abundant EV markers, they do not play a major, general role in determining EV protein composition in MCF7 cells (Fan et al., 2023). Consistent with this, studies using CD81 knockout lymphoblasts showed that the overall protein composition, EV size, and release levels of EVs were largely unaffected, with over 80 % similarity in cargo compared to wild-type EVs (Perez-Hernandez et al., 2013). In sum, CD81 can tolerate certain C-terminal fusions without severely disrupting EV incorporation, suggesting that with careful design, these tetraspanins can be effectively used for EV engineering while retaining key aspects of their natural behavior. While prior studies demonstrate that certain C-terminal fusions to CD81 do not impair EV secretion or trafficking, it remains possible that the specific fusion of mNeonGreen could alter CD81 functional properties. This potential limitation should be considered when interpreting EV loading or targeting outcomes in this system.

In summary, the AstroGreen transgenic mouse makes it possible to monitor the secretion of EVs by astrocytes in the CNS in distinct brain regions, capture and track their distribution, and show their uptake *ex vivo,* and potentially *in vivo*. In this study, we have characterized and shown a proof-of-concept in using this genetically engineered model to follow and assay cargo delivery of cell-specific EVs. Next, functional assays following cellular changes upon EV cargo delivery are needed to fully encipher the role of the EV in communication between astrocytes and tumor cells. Many studies are now focusing on the role of astrocytes in neurodegenerative disease progression (Lee et al., 2022), such as Alzheimer's disease (Monterey et al., 2021; Habib et al., 2020), Huntington's disease (Khakh and Goldman, 2023) and Parkinson's disease (Miyazaki and Asanuma, 2020), and in particular astrocyte interactions with other cell types and their effects on pathologic progression (Huffels et al., 2023; Gotoh et al., 2023). Crossbreeding of the established AstroGreen mouse model with existing mouse models that reflect pathologies of the CNS could help in understanding the effect of astrocyte-derived EVs in brain diseases. Therefore, the AstroGreen transgenic mouse model will be of great interest to study astrocyte function and communication in a neurological pathology context.

## 4. Limitations of the study

Although the GFAP promoter can cause leaky Cre expression in neural progenitor cells (Sofroniew, 2012; Zhuo et al., 2001; Casper and McCarthy, 2006) and Schwann cells (McKeon and Benarroch, 2018), the GFAP-Cre reporter model is effective in astrocytes, with no Cre activity observed in postnatal or adult neural stem cells (Gregorian et al., 2009). Given that our mouse model is based solely on the robust expression of GFAP, this poses a limitation to this model. GFAP expression varies by age, brain region, phenotype, and physiological condition. Additionally, GFAP expression is affected by the developmental stage of the mouse brain, hence studies aimed at studying astrocytes in the context of the developing brain may be limited. Future studies should take into consideration both the strengths and limitations of using the GFAP promoter to target astrocytes specifically and broadly.

Reactive astrocytes show increased expression of GFAP which would argue that the AstroGreen transgenic model could be most useful in pathological studies. Alternatively, the Exomap1^+/+^ mouse could be crossed with the Aldh1l1-Cre reporter mouse (B6;FVB-Tg(Aldh1l1-cre) JD1884Htz/J; strain: 023748) which might have an advantage when studying astrocytes in a more homeostatic setting. Lastly, using the astrocyte specific inducible model (GFAP-CreERT mouse line), where Cre recombinase is fused to a triple mutant form of the human estrogen receptor (CreERT), could possibly reduce the association with astrocytosis since Cre would not be constantly expressed in the astrocytes. The expression of this tamoxifen-inducible Cre recombinase driven by a human GFAP promoter would allow Cre expression only upon tamoxifen induction following loxP recombination (Ma et al., 2018). Of note, this is dependent on the penetration and distribution capacity of tamoxifen throughout the brain after intraperitoneal administration. Future studies should take into consideration both the strengths and limitations of using the GFAP promoter to target astrocytes specificity and broadly.

In addition, we acknowledge that the interstitial space between tissue can potentially compromise the extracellular environment and fragmentation of cell membranes could contaminate the isolated BdEVs. In this study, we used a recently optimized method to enrich EV populations from brain tissue, previously described by Vella et al., 2017, this BdEV isolation method has been used by several groups (Rufino-Ramos et al., 2023; Huang et al., 2022; Breyne et al., 2022; Su et al., 2021; D'Acunzo et al., 2022). Despite enrichment of BdEVs based on traditional EV markers, including CD9 and FLOT-1, and undetectable levels of the CNX marker, further steps could be taken to eliminate the co-isolation of, for example, DNA originating from nuclei disruption, protein contaminants in BdEV preparations and non-EV particles that overlap EVs in size and morphology. We therefore recognize the possibility that our BdEVs samples could contain some non-EV particles generated by our protocol which might account for the observed substantial differences between BdEVs and MdEVs. Specifically, we found that BdEVs were distinct from MdEVs in that their mNeonGreen fluorescence was ~30-fold higher, their levels of mRNA were 10-fold higher for HsCD81mNG and 200-fold higher for Cre, their immunostaining with anti-HsCD81mNG antibodies was ~100-fold higher, and ~ 50 % of BdEVs could not be captured on anti-HsCD81 antibody beads, likely because of inefficient pulldown rather than intrinsic biological properties. Notably, primary cultured astrocytes isolated from neonatal pups are selectively derived from the cortices which results in a more homogenous MdEV population. In contrast the BdEVs are derived from slices throughout the entire adult brain, containing multiple brain regions resulting in a more mixed BdEV sample, which in part might account for the observed differences between BdEVs and MdEVs.

Future studies are needed to elucidate the molecular mechanisms that underly these substantial differences between tissue-derived and cell-derived EVs and raise the possibility that there may be limitations to tissue-derived EV isolation methods (Brenna et al., 2021; Crescitelli et al., 2021).

## 5. Methods

### 5.1. Experimental model and study participant details

All animal experiments were conducted under the oversight of the Massachusetts General Hospital Institution Animal Care and Use Committee (IACUC) and all experiments were performed to conform to all relevant regulatory standards (protocol 2009 N000054). C57BL/6 J was obtained from Charles River Labs. The B6.Cg-Tg(Gfap-Cre)77.6Mvs/2 J (strain: 024098) (Gregorian et al., 2009) and B6.Cg-Gt(ROSA)26Sor^tm9 (CAG-tdTomato)Hze^/J (strain: 007909), were obtained from the Jackson Laboratory. Exomap1 mice were described previously (Fordjour et al., 2023). The Exomap1 mice were crossed with the Gfap-Cre transgenic mice to generate AstroGreen transgenic mice. Mice were maintained with unlimited access to water and food under a 12-hour (hr) light/dark cycle. Experiments were performed on mice aged 8–10 weeks. Sample size per experiment is listed in the figure legends, using equal number of males and females. Allocation to experimental groups was done randomly.

### 5.2. Method details

#### 5.2.1. Genotyping

Genotyping was performed using genomic DNA isolated from mouse tail and PCR, using the KAPA HotStart Mouse Genotyping Kit (No. KK7352; Kapa Biosystems), primers for tdTomato-HsCD81 and GFAP-Cre, and primers to distinguish homozygous from heterozygous mice (Table 1). DNA extractions were performed in 100 μl using KAPA Express Extract to digest the tissues followed by a 15-min lysis step. Afterwards, DNA was diluted 10-fold in 10 mM Tris-HCl, pH 8.0. PCR was performed using KAPA2G Fast DNA Polymerase in KAPA2G Fast Genotyping Mixes under the following cycling conditions: initial denaturation at 94 °C for 3 min; denaturation at 94 °C for 30 s; annealing at 62 °C for GFAP, 60 °C for CD81 and 60 °C for homozygous-HsCD81 for 1 min (repeated 10 X); 94 °C for 30 s; 62 °C for 30 s then extension at 72 °C for 1 min (repeated 28×). The final extension is at 72 °C for 5 min. The expected amplicon sizes for each set of primers are tdTomato-HsCD81 500 bp; GFAP-Cre 400 bp; GFAP wild-type and GFAP homozygous should not show any band (Fig. S1A–B).

#### 5.2.2. Cells

The mouse CT-2A cell line was provided by The National Cancer Institute (NCI). CT-2A cells were cultured in a 5 % CO_2_ humidified incubator at 37 °C in Dulbecco's modified Eagle's medium (DMEM, Corning) with 10 % fetal bovine serum (FBS, Gemini Bioproducts, West Sacramento, CA) and 1 % penicillin (100 units/ml) /streptomycin (100 mg/ml) (P/S). Cells were checked regularly for mycoplasma infection using the PCR Mycoplasma Detection Kit (G238; ABM). The plv-cmv-loxp-bfp-loxp-iRFP plasmid construct (BFP/iRFP) (GenScript) was obtained from Dr. Miller's Laboratory (MGH). CT-2A cells were stably transduced with a lentiviral vector to express the blue fluorescent protein (BFP)/near-infrared fluorescent protein (iRFP).

#### 5.2.3. Mixed glial cultures

Mixed glial cultures were isolated from cerebral cortices of P0 to P4 AstroGreen and Exomap1 pups, as described (Zelenka et al., 2022). Meninges were removed, and cortical cells were dissociated using 0.05 % Trypsin/EDTA (Corning) followed by single-cell suspension using 100 mm and 40 mm cell strainers (BD Falcon). Cells were cultured in DMEM with 20 % FBS, 1 % P/S, and 10 ng/ml M-CSF (Gibco) on poly-D-lysine (PDL; Sigma-Aldrich;10 mg/ml) pre-coated T-75 culture flasks for 10–15 days. Primary microglia were removed from confluent mixed glial cultures by gentle shaking on an orbital shaker for 1 h at 180 rpm. Astrocytes were collected in the medium by centrifugation at 300 ×*g* after further shaking overnight at 230 rpm.

#### 5.2.4. Intracranial tumor implantation

Adult mice were anesthetized using 2.5 % isoflurane in 100 % oxygen *via* a nose cone. A total of 1 × 10^5^ CT-2A-BFP/iRFP(GenScript) cells stably expressing Firefly luciferase (Fluc) were suspended in 2 μl Opti-MEM (Gibco). The cells were then injected into the left striatum of C57BL/6 J mice using a Hamilton syringe (Sigma-Aldrich) and automatic stereotaxic injector (Stoelting) with a flow rate of 0.2 ml/min. In reference to bregma, three stereotactic coordinates aiming for the left striatum with respect to the skull were chosen: anterior-posterior (AP) = 0.5 mm, medial-lateral (ML) = 2.0 mm, and dorsal-ventral (DV) = 2.5 mm.

#### 5.2.5. Tissue digestion

Tumor-bearing mice (CT-2A-mCherry or CT-2A-BFP/iRFP both cell lines expressing Fluc) were transcardially perfused with 50 ml Dulbecco's phosphate-buffered saline (DPBS) after intraperitoneal injection of a mixture 100 μl ketamine (17.5 mg/ml) (Patterson Veterinary) and xylazine (2.5 mg/ml) (Patterson Veterinary). Tumor Tissue Dissociation Kit (Miltenyi Biotec) was used to process the brain into a single-cell suspension. The ipsilateral hemisphere of the brains was placed into a GentleMacs C-tube (Miltenyi Biotec) with 2.35 ml RPMI 1640 containing enzymes D (100 μl), R (30 μl), and A (3.5 μl) and incubated for 40 min at 37 °C. According to the manufacturer's protocol, the brains were dissociated using the gentle MACS Dissociator (Miltenyi Biotec). Samples were run through a 70 μm filter to obtain a single-cell suspension. Myelin removal was achieved using magnetic separation with anti-myelin beads (Miltenyi Biotec). The final cell suspension was resuspended in 1× DPBS without calcium (Ca^2+^) or magnesium (Mg^2+^) (Corning), supplemented with 2 mM EDTA (Thermo Fisher) and 0.5 % BSA (Sigma). Single cells were further processed either using FACS to sort BFP and iRFP positive tumor cells to analyze Cre-driven recombination events using expression analysis by RT-qPCR or by flow cytometry analysis to quantify the percentage of BFP or iRFP tumor cells.

#### 5.2.6. RT-qPCR

cDNAs for gene expression analysis by RT-qPCR were prepared using the SuperScript VILO cDNA Synthesis Kit (Invitrogen). qPCR mix was prepared following the manufacturing protocol of Power SYBR Green PCR Master Mix (Applied Biosystems). qPCR was performed using the QuantStudio 3 PCR system (Applied Biosystems). The cycling conditions used were 50 °C for 2 min, 95 °C for 10 min, 40 cycles at 95 °C for 15 s, and 60 °C for 1 min. All qPCR reactions were done in triplicate and normalized to β*-Actin* mRNA levels. Primers were obtained through http://www.origene.com/ or custom-designed with SnapGene^®^ software (Table 2).

#### 5.2.7. Immunofluorescence imaging

Cells or brain slices (12 μm) were fixed with 4 % PFA and rinsed twice in PBS for 5 min each. Blocking was achieved by using 5 % BSA and 0.1 % Tween-20 in PBS (PBS-T) for 1 h. Cells or brain slices were then incubated with the primary antibodies – mNeonGreen (1:400, Chromotek #32F6); GFAP (1:400, Invitrogen #13–0300); Aldehyde Dehydrogenase 1 Family Member A1 (ALDH1a1) (1:400, Abcam #ab227964); ACSA-2 (1:400, Miltenyi Biotec #130–099–138); HsCD81 (1:400, Abcam #ab219209); IBA1 (1:400, FujiFilm Wako #019–19,741); TMEM119 (1:400, Invitrogen #Ma5–50,522); NeuN (1:400 Abcam #ab177487); and OLIG2 (1:400 Abcam #ab109186) at 4 °C overnight. Cells or brain slices were rinsed three times in PBS-T for 5 min each. The appropriate secondary antibody (goat anti-rabbit; goat anti-rat; goat anti-mouse Invitrogen) was diluted 1:400 in PBS-T and incubated for 1 h in the dark at RT. After washing with PBS for 5 min, coverslips were transferred to microscope slides (Fisherbrand), and brain slices were mounted with a droplet of mounting medium with DAPI (Vectashield, Vector Labs). Fluorescence microscopy images were acquired with the Zeiss LSM 710 confocal microscope or the Nikon W1-SoRa confocal microscope. Scans of the sagittal and coronal sections of the AstroGreen brain were scanned with the Axio Scan.Z1 (Zeiss). All images were processed using ImageJ 1.49v and ImageJ2 (FIJI) version 2.9.0 software.

#### 5.2.8. EVs enrichment from brain tissue

The mouse brain was collected after PBS perfusion and stored at −80 °C, until further processing. The frozen tissue was sliced sagittal (roughly 1/3 of the brain) on ice to generate 1–2 cm long, 2–3 mm wide tissue sections. Further processing of sections to obtain EVs was done based on protocols reported previously (Vella et al., 2017; Rufino-Ramos et al., 2023; Huang et al., 2020; Su et al., 2021). The tissue pieces from each sample were weighed and incubated with 50 U/ml collagenase type D (Roche) in DMEM medium (at a ratio of 8 μl/mg tissue) in a shaking incubator platform at 50 rpm (RT for 20 min). After 10 min of incubation, samples were twice resuspended and incubated for another 10 min, followed by the addition of protease and phosphatase inhibitors (Halt) in PBS at a final concentration of 1×. The digested brain extracts were subjected to the following centrifugation steps at 4 °C: 300 ×g for 5 min, 2000 ×g for 10 min, and 10,000 ×g for 30 min. One ml supernatant was then incubated with 5 μl DNase (stock solution of 10 mg/ml, Zymo research) for 10 min and then filtered through a 0.22 μm filter (Millex). The 10,000 ×g supernatant was loaded onto qEV original size exclusion chromatography (SEC) columns (SP1, IZON Science) and 24 fractions (500 μl each) were collected by elution with PBS using the Automatic Fraction Collector (IZON) according to the manufacturer's protocol.

#### 5.2.9. SEC for EV isolation

Primary-derived astrocytes were cultured for 72 h at 90 % confluency in T75 flasks. In total 15 ml EV-depleted-20 % FBS (FBS was depleted from EV by 16 h of ultracentrifugation at 160,000 ×*g*) conditioned medium were collected per flask and centrifuged at 300 ×*g* for 10 min to remove cellular debris. The medium was then concentrated with UFC9100 Amicon^®^ Ultra-15 Centrifugal filters to a final volume of 500 μl (spun at 6000 ×*g* for 15 min). Concentrated media was applied to IZON qEV original size exclusion columns according to the manufacturer's protocol followed by flushing the column with 1× PBS. Fractions 7 to 11 were collected and up concentrated with 100 kDa Amicon^®^Ultra-0.5 Centrifugal filters to a final volume of 100 μl. To characterize the full profile of the primary astrocyte secreted particles, fractions 7 to 30 were collected and further analyzed for fluorescence intensity (Excitation 500 nm/Emission 520 nm) in the plate reader - Synergy H1 Hybrid Multi-Mode Reader (BioTek). EV fractions 7–11 were characterized by size distribution analysis using nanoparticle tracking analysis (NTA), with screen gain set at 7.0 and camera level 12.0.

#### 5.2.10. Nanoparticle tracking analysis

EVs were diluted (1:10; 1:50; 1:100) in filtered PBS before analysis using Nanoparticle Tracking Analysis Version 2.2 Build 0375 instrument (NanoSight). We used variable dilutions for our NTA measurements as sample concentrations differ between biological replicates or experimental conditions. Using a fixed dilution assumes uniform EV levels across samples, which is rarely the case in practice. Variable dilutions are required to bring all samples into the optimal detection window of the NTA device. Overly concentrated samples result in overlapping particle tracks and hindered Brownian motion, compromising size and concentration measurements. Conversely, overly diluted samples may fall below the detection threshold, increasing variability and the likelihood of detecting background noise or contaminants.

Particles were measured by the acquisition of 3 videos of 30 s and the number of particles (30–800 nm) was determined using NTA Software 2.2. The following photographic conditions were used: frames processed (1498 of 1498 or 1499 of 1499); frames per second (f/s) (24.97 f/s or 24.98 f/s); calibration (190 nm/pixel); and detection threshold (6 or 7 multi). The number of particles per frame was within the recommended range of 20–100 particles/frame for NanoSight NS300, detection threshold 4, and cameral level 10.

#### 5.2.11. Particle fraction fluorescence analysis and treatment with Proteinase K and/or Triton X-100

To analyze if mNeonGreen is associated with and protected by the membrane of the EVs, SEC fractions of BdEVs were treated with proteinase K (New England Biology), degrading the proteins inside of the EV membrane and Triton X-100 (USB), which disrupted the membrane of EVs. Three conditions were compared; 1) non-treated BdEVs (EV fractions without proteinase K and/or Triton X-100; 2) BdEV SEC fractions treated with proteinase K only; 3) BdEV SEC fractions treated with both proteinase K and Triton X-100.

For proteinase K treatment, 100 μl of each fraction (7–30) from BdEVs were incubated with 1 % Proteinase K in a 96-well plate. For proteinase K + Triton X-100 treatment, 100 μl of each SEC fraction were incubated with both 1 % Triton X-100 (USB) and 1 % Proteinase K (New England Biology) in a second 96-well plate. Non-treated and treated SEC fractions were then incubated for 1 h at 37 °C and analyzed for mNeonGreen fluorescence intensity (Excitation 500 nm/Emission 520 nm) in the plate reader - Synergy H1 Hybrid Multi-Mode Reader (BioTek).

#### 5.2.12. CD81 capture beads incubation

Brain-derived EVs (BdEVs) were incubated with human CD81 capture beads (Sapphire #81CB-25) to enrich for HsCD81 EVs on a shaker for 48 h at 4 °C. After the EVs were pulled down, the supernatant was removed and beads with bound EVs were washed with PBS twice. Beads and EVs were incubated with FC-block (1:100 anti-mouse CD16/32, BioLegend, #101319) for 10 min on ice. After washing, samples were resuspended in 100 μl FACS buffer (0.5 % BSA in DPBS and incubated with anti-human CD81 PE-fluorophore (TAPA-1; 1:100, BioLegend #349505) or the isotype control PE fluorophore mouse IgG1 (1:100 BioLegend #400113) for 40 min on a shaker at 4 °C. Beads and EVs were spun down and washed twice with PBS. Beads with bound EVs were resuspended in 200 μl FACS buffer and the PE and FITC fluorophores were lysed for western blot analysis or measured by SORP 5 Laser BD LSRFortessa X-20 and analyzed by FlowJo (https://www.flowjo.com). BdEVs bound to HsCD81 beads were imaged using fluorescence microscopy images acquired with the Zeiss LSM 710 confocal microscope and processed using ImageJ 1.49v software.

#### 5.2.13. Western blot

Before western blot analysis cells and EVs were lysed in RIPA buffer with protease inhibitors (Sigma). Protein concentration was determined using Pierce BCA protein assay (Thermo Fisher) and equal amounts (20 μg) were loaded and resolved on 10 % SDS-PAGE gels (Thermo Fisher). After transfer onto nitrocellulose membranes, they were blocked for 1 h in 5 % milk in water and probed at 4 °C overnight for Calnexin (CNX) (1:1000, Santa Cruz, #sc-46,669); mNeonGreen (1:1000, Chromotek #32F6); HsCD81 (1:20, Abcam #ab209129); FLOT-1 (1:1000, Abcam #ab133497); GFAP (1:1000, Invitrogen #13–0300); CRE (1:1000, Abcam #ab188568); CD9 (1:500, Santa Cruz #sc-13,118); Connexin-43 (CX43) (1:1000, Cell Signaling #3512); GLUT-1 (1:1000, Cell Signaling #12939); Aquaporin-4 (AQP4) (1:1000, Cell Signaling #59678); and housekeeping β-ACTIN (1:1000, Santa Cruz, #sc-1616). Samples were incubated with secondary antibody for 1 h at RT. Enhanced chemiluminescence ECL (Thermo Fisher) with Femto (Thermo Fisher) was used for immunoblotting with Anti-Rabbit IgG (1:5000, Sigma); Anti-Goat IgG (1:5000, Abcam) and Anti-mouse IgG (1:5000, Thermo Fisher) corresponding to the primary antibody used. The Protein ladder (Biorad #1610374) was used to determine molecular weight sizes.

#### 5.2.14. Flow cytometry

Astrocytes were isolated using anti-ACSA-2 microbeads (Miltenyi Biotec) after isolation using the LS column (Miltenyi Biotec) and separated by the QuadroMACS^™^ Separator (Miltenyi Biotec). The ACSA-2 positive astrocytes were then incubated for 10 min on ice in FACS buffer (DPBS without Ca^2+^ or Mg^2+^ and 0.5 % BSA) supplemented with TruStain fcX (anti-mouse CD16/32, BioLegend, #101319, clone 93, 1:100). Cells were stained for 10 min with LIVE/DEAD^™^ Fixable Blue Dead Cell Stain Kit for UV excitation (Invitrogen #L23105, 1:300). Cells were stained with anti-ACSA-2-PE (Miltenyi Biotec #130–116–246, 1:100) and anti-GFAP-APC (Thermo Fisher #50–9892–82, 1:100) for 30 min at RT. Finally, cells were washed with 1 ml DPBS (without Ca^2+^ or Mg^2+^) with 0.5 % BSA and 2 mM EDTA and were centrifuged at 300 ×*g* for 10 min, resuspended in 0.5 % BSA, 2 mM EDTA in DPBS (without Ca^2+^ or Mg^2+^) and passed through a 35 mm nylon mesh strainer (BD Falcon).

To perform flow cytometric analysis on both BdEVs and MdEVs, EVs were first incubated with human CD81 capturing microbeads (Sapphire, Cat#: 81CB-25) for 48 h. Microbeads were washed with PBS and blocked for 10 min with TruStain fcX (1:100). Microbeads were washed once with PBS and incubated with PE anti-human CD81(TAPA-1) (BioLegend, Cat#: 349505; 1:100) antibody or the PE Mouse IgG control (FC) (BioLegend, Cat#: 400113; 1:100) for 40 min at RT on a shaker. After staining, beads were washed two times with PBS and 100 μl FACS buffer was added before analysis.

To analyze the percentage of BFP and iRFP cells of tumor-bearing mice, brains were harvested and processed according to the tissue digestion protocol at day 14 post-tumor implantation. CD45-positive cells were isolated using the CD45 microbeads (Miltenyi Biotec). To separate CD45 negative cells from the CD45 positive cell population, single cell suspension was applied on the LS column (Miltenyi Biotec) and separated using the QuadroMACS^™^ Separator (Miltenyi Biotec). The CD45 positive cells were centrifuged at 300 ×*g* for 10 min, resuspended in 0.5 % BSA, 2 mM EDTA in DPBS (without Ca^2+^ or Mg^2+^), and passed through a 35 mm nylon mesh strainer (BD Falcon).

Samples were measured by SORP 5 Laser BD LSRFortessa X-20 and analyzed by FlowJo (https://www.flowjo.com).

BdEVs purified from AstroGreen mice and media-derived EVs (MdEVs) isolated from cultured media incubated with primary astrocytes were diluted 1:60 and incubated on the ExoView Tetraspanin Chip for 16 h at RT, following manufacturer's guidelines (NanoView Biosciences). After washing the chips three times in PBS for 3 min each on an orbital shaker at 500 ×g, they were incubated with the ExoView Tetraspanin labeling antibodies; HsCD81 (1:50 in PBST with 2 % BSA) for 1 h. The chips were washed again with PBS three times for 3 min each in an orbital shaker at 500 ×g and imaged and analyzed using the ExoView R100 reader.

#### 5.2.15. EV transfer in vitro

CT2A-BFP/iRFP expressing cells were transfected with 400 ng Cre expressing plasmid. Cre was cloned into the pCS2-Cas9 plasmid (Addgene plasmid #47322) (Gagnon et al., 2014) and diluted in Opti-MEM (Gibco) medium containing Lipofectamine 2000 (Thermo Fisher). In parallel CT2A-BFP/iRFP cells were incubated with either 1 × 10^9^ Exomap1 BdEVs or AstroGreen BdEVs for 5 days. Cells were either mounted with a mounting medium containing DAPI (Vectashield, Vector Labs) to view fluorescence or RNA was extracted following the manufacturing protocol of the Quick-RNA Miniprep Kit (Zymo Research). The floxed allele was analyzed with RT-qPCR analysis using BFP unfloxed and iRFP floxed primers designed with SnapGene^®^ software and are specified in (Table 2).

#### 5.2.16. Quantification and statistical analysis

Bar graphs were made in GraphPad Prism 9.5.1. Error bars show the mean ± standard error of the mean (SEM). A one-way ANOVA, two-way ANOVA and multiple *t*-tests were applied to determine if conditions significantly differed. Statistical significance was specified as *p* < 0.05. Sequences and plasmid constructs were analyzed with Snapgene software version 6.0.2.

## Supplementary Material

1

## Figures and Tables

**Table 1:** Primers genotyping.

Gene	Primer	Sequence (5′-3′)

**tdTomato-HsCD81**	Forward	GATGGGAAGCAAGCACAGAAAGACTC
	Reverse	GATTCGTTGTTTGCGGGTCGTG
**Gfap-Cre**	Forward	TCCATAAAGGCCCTGACATC
	Reverse	TGCGAACCTCATCACTCGT
**Homozygous-HsCD81**	Forward	TGGAGGAGGACAAACTGGTCAC
	Reverse	TTCCCTTTCTGCTTCATCTTGC
**Homozygous-Cre**	Forward	CAAATGTTGCTTGTCTGGTG
	Reverse	GTCAGTCGAGTGCACAGTTT

**Table 2:** Primers RT-qPCR.

Gene	Primer	Sequence (5′-3′)

***β-Actin***	Forward	GTGACGTTGACATCCGTAAAGA
	Reverse	GCCGGACTCATCGTACTCC
***HsCD81mNG-1***	Forward	CGATGCAAATAACGCCAAGGC
	Reverse	TAAAGTTTACCGCTGAAGAGGTCATCA
***HsCD81mNG-2***	Forward	CATCTCGGGTATGGCTTCCATCAG
	Reverse	TGCGATGGACTTGGTATCCG
***GfGap***	Forward	TGCCACGCTTCTCCTTGT
	Reverse	TTCAGCTGCCAGCGCC
***Aldh1a1***	Forward	GGAATACCGTGGTTGTCAAGCC
	Reverse	CCAGGGACAATGTTTACCACGC
***Slac1a3***	Forward	GCGATTGGTCGCGGTGATAATG
	Reverse	CGACAATGACTGTCACGGTGTAC
***Iba1***	Forward	TCTGCCGTCCAAACTTGAAGCC
	Reverse	CTCTTCAGCTCTAGGTGGGTCT
***Tmem119***	Forward	ACTACCCATCCTCGTTCCCTGA
	Reverse	TAGCAGCCAGAATGTCAGCCTG
***P2ry12***	Forward	CATTGACCGCTACCTGAAGACC
	Reverse	GCCTCCTGTTGGTGAGAATCATG
***Cre***	Forward	CGGTCGATGCAACGAGTGAT
	Reverse	CAGGTATGCTCAGAAAACGCC
***tdTomato***	Forward	TTCATGTACGGCTCCAAGGC
	Reverse	ACCTTGTAGATCAGCGTGCC
	Reverse	AGCAGTGAGGTCAGGCTTGGAA
***iRFP Floxed***	Forward	ACCGTCAGATCGCCTGG
	Reverse	CAAGAGGTCAGGCTGCCT
***BFP Unfloxed***	Forward	ACCGTCAGATCGCCTGG
	Reverse	CCTTAATCAGCTCGCTCATGGT

**Table 3:** Quantification NTA of EVs per brain region.

Brain region	AstroGreen		Exomap1	
		
	Particles/ml	Dilution	Particles/ml	Dilution

**Olfactory bulbs**	1.29e+08	1:10	1.40e+08	1:10
**Cortex**	3.70e+07	1:100	1.28e+08	1:100
**Cerebellum**	3.94e+08	1:10	1.36e+08	1:10
**Hippocampus**	1.35e+08	1:50	1.16e+09	1:50
**Thalamus**	7.39e+07	1:50	1.40e+08	1:10
**Medulla & Pons**	7.98e+07	1:50	2.57e+07	1:50
**Brainstem**	3.09e+07	1:50	2.00e+08	1:50

## Data Availability

The authors confirm that the data supporting the findings are available within the article and its supplementary material. Requests for further information and resources should be directed to and will be fulfilled by the lead contact, Erik R. Abels (e.r.abels@lumc.nl). The authors confirm that the data supporting the findings are available within the article and its supplementary material. Raw data (RT-(q)PCR, flow cytometry, immunofluorescent imaging and western blot) that support the findings reported in this study are available upon request. In this study no new datasets of a standardized datatype are generated.• No original code has been written for this manuscript.• Any additional information required to analyze, or questions associated with the data are available upon request.

## Appendix A. Supplementary data

Supplementary data to this article can be found online at https://doi.org/10.1016/j.mcn.2025.104051.
