# Supplementary material for: AstroGreen transgenic mouse illuminates the trafficking of astrocyte-derived extracellular vesicles
Source: Mol Cell Neurosci. Author manuscript; Available in PMC 2026 Sep 9. (PMC13553430; doi:10.1016/j.mcn.2025.104051)
Supplement: Supplementary file 1 [file NIHMS2184673-supplement-1.pdf]

Supplemental information

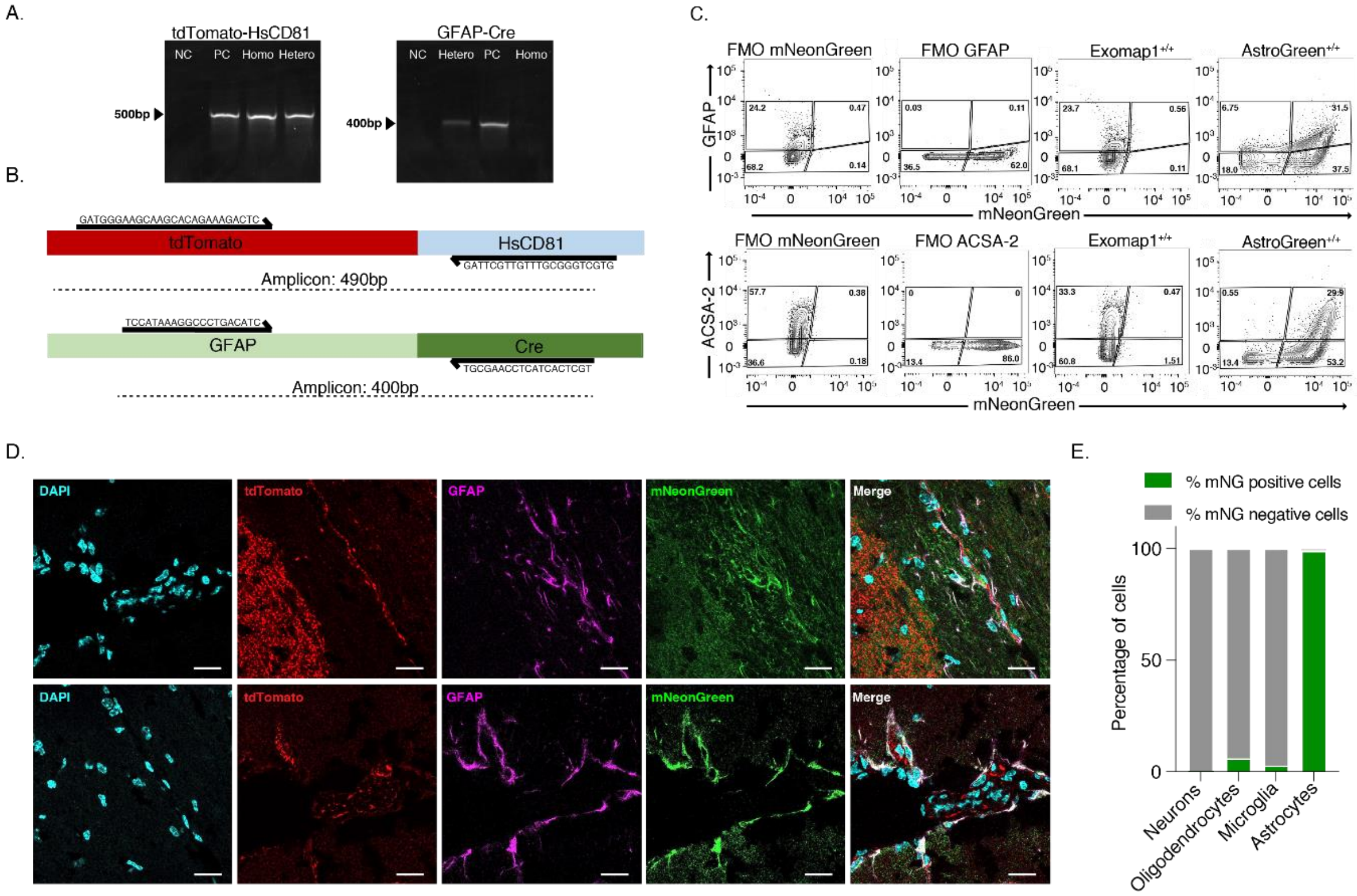

Figure S1. Characterization of the AstroGreen$^{+/+}$ mouse brain.

(A) Genotype result to screen for GFAP-HsCD81$^{+/+}$ (AstroGreen$^{+/+}$) mice (NC = Negative control - H2O; PC = Positive control – DNA from the homozygous parent; hetero = Heterozygous; Homo = Homozygous) two primer sets were used tdTomato-HsCD81 with an expected amplicon of 490bp, and GFAP-Cre amplifying a 400bp band and GFAP wild-type and GFAP homozygous should not show any band (**Table 1**). (B) Schematic display of primers tdTomato-HsCD81 and GFAP-Cre used to perform genotyping assay. (C) Wild-type C57BL/6J mice were used to determine fluorescence minus one (FMO) control to specify gating for mNeonGreen. AstroGreen$^{+/+}$ mice were used to specify GFAP and ACSA-2 FMO gates. Gates were determined in brains of ExoMap$^{+/+}$ and AstroGreen$^{+/+}$ mice. (D) Immunohistochemistry around the ventricle and hippocampus area showing representative images of separate channels of the AstroGreen$^{+/+}$ brain DAPI, tdTomato, mNeonGreen, GFAP (goat anti-rat Alexa Fluor 647) and merge (Magnification 60x; scale bar 50 µm). (E) Quantification of (n=5-6) representative IHC images of the percentage of mNeonGreen positive and negative cells. For NEUN^{+} cells (neurons), 1 out of a total of 257 cells co-stained for mNeonGreen (0.39%), for OLIG2^{+} (oligodendrocytes) 9 out of a

total of 151 (5.96%) were double positive for mNeonGreen, for IBA1$^+$ (microglia) 3 out of a total of 106 cells (2.83%) showed colocalization of mNeonGreen, while 363 out of 367 (98.91%) GFAP$^+$ (astrocytes) stained positive for mNeonGreen.

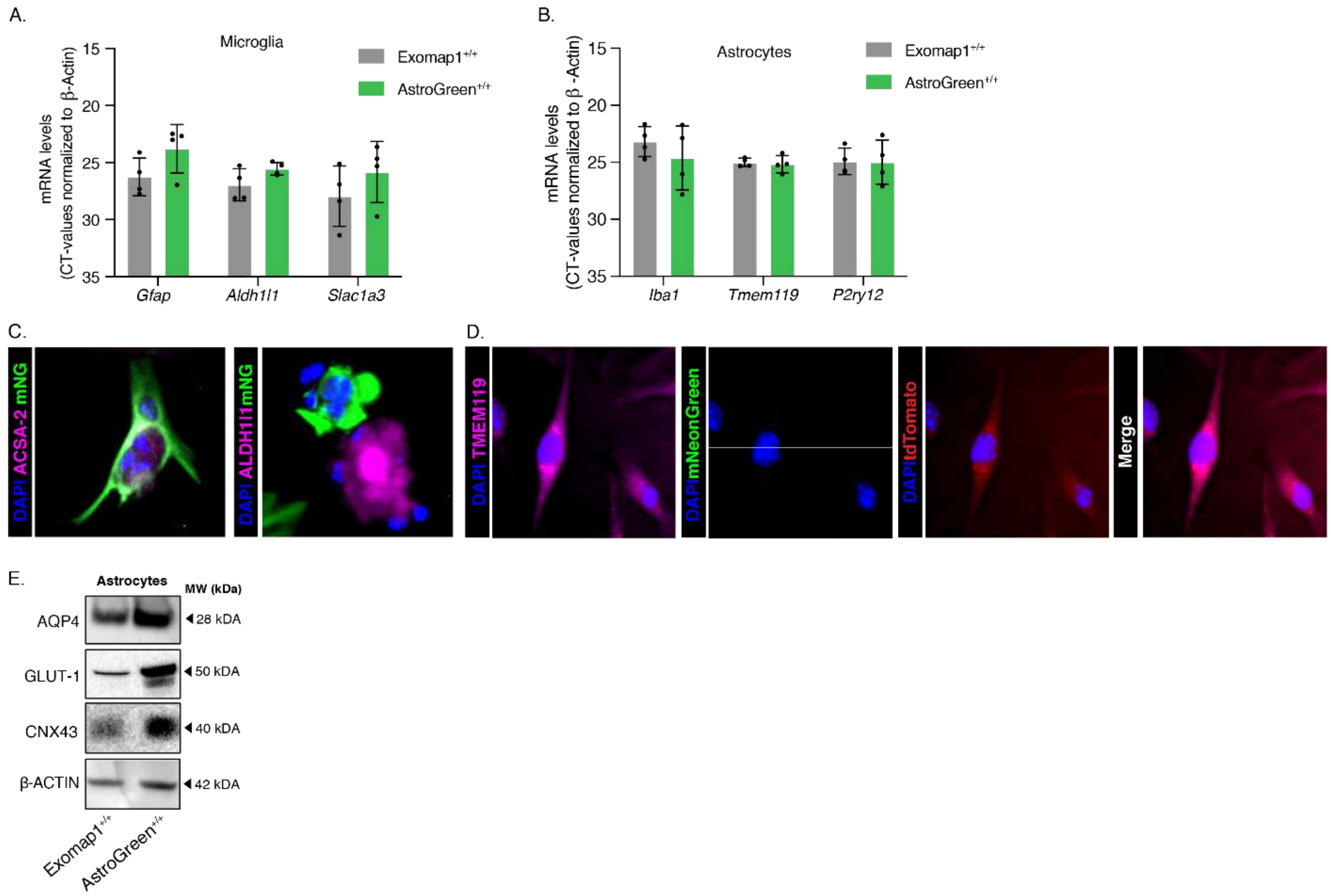

Figure S2. *In vitro* Characterization of AstroGreen$^{+/+}$ derived astrocytes.

(A) Expression levels of astrocyte specific markers show Exomap1 and AstroGreen primary derived astrocytes express *Gfap*, *Aldh1a1* and *Slac1a3* mRNA with levels normalized to the housekeeping gene *β-Actin*. (B) Microglia isolated from the AstroGreen mixed glial cell cultures express microglia specific markers, *Iba1*, *Tmem119* and *P2ry12* mRNA with levels were normalized to the housekeeping gene β-Actin. Data represent three independent experiments and are presented as the mean with SEM (error bars). (C) AstroGreen$^{+/+}$ cultured astrocytes expressing mNeonGreen are positive for ACSA-2 (goat anti-rat Alexa Fluor 647) (left) but negative for ALDH1A1 (goat anti-rabbit Alexa Fluor 647) (right) (Magnification 60x; scale bar 50 µm). (D) Immunohistochemistry of microglia derived from AstroGreen$^{+/+}$ mixed glial cell cultures stained for TMEM119 (goat anti-rabbit Alexa Fluor 647) were negative for mNeonGreen (Magnification 60x; scale bar 50 µm). (E) Protein expression analysis show presence of astrocyte markers AQP4, GLUT-1 and CNX43 compared to β-ACTIN for both AstroGreen$^{+/+}$ and Exomap1$^{+/+}$ primary cultured astrocytes. Data represent one experiment.

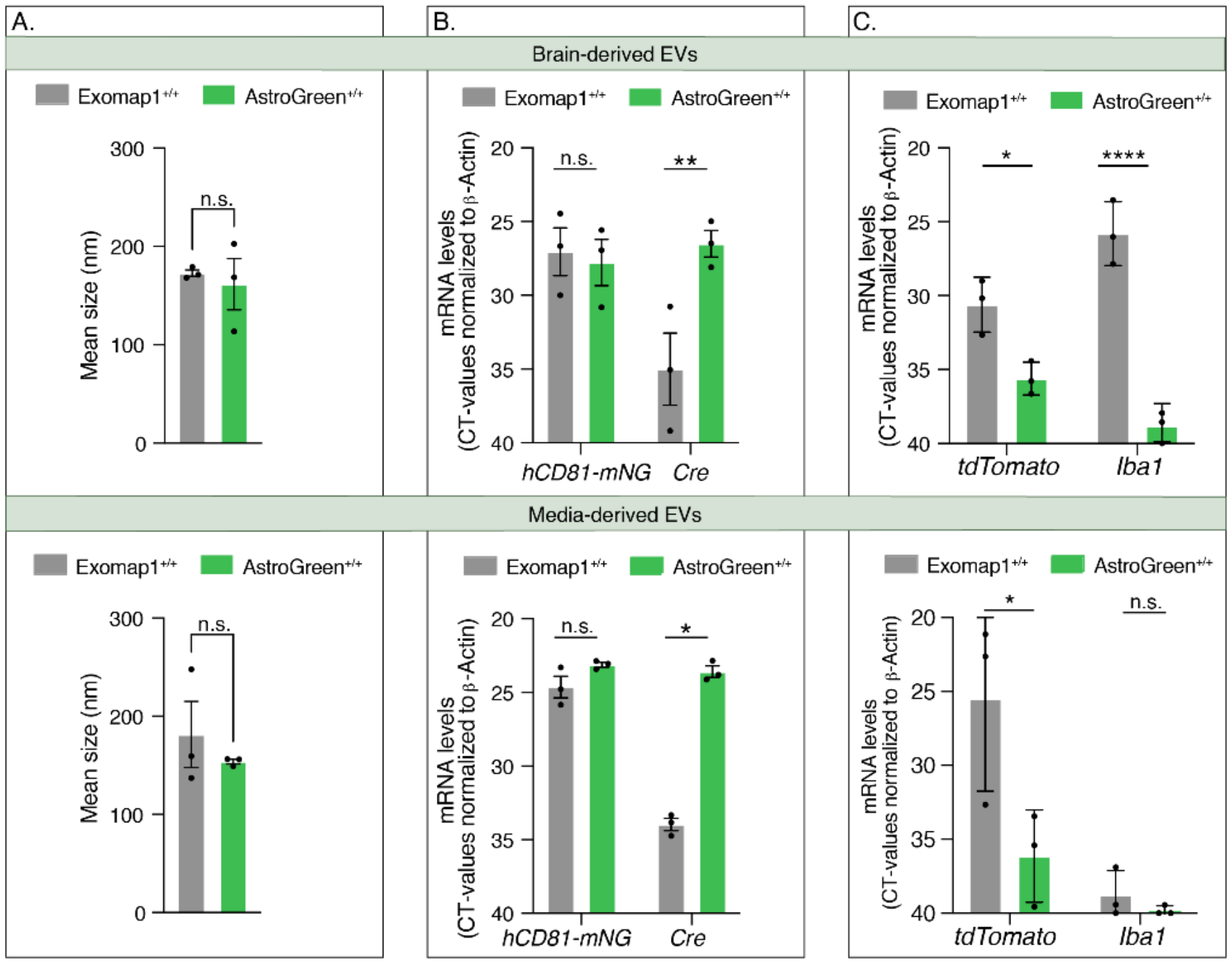

Figure S3. Quantification of AstroGreen$^{+/+}$ and Exomap1$^{+/+}$ BdEVs and MdEVs.

(A) Nanoparticle tracking analysis showing mean size (nm) of Exomap1 and AstroGreen BdEVs (top) and MdEVs (bottom). No significant differences were observed. Data is plotted as the mean with (SEM) error bars. Unpaired t test, not significant (n.s.). (B) Expression levels for *Cre* were significantly increased in AstroGreen$^{+/+}$ compared to Exomap1$^{+/+}$ for both BdEVs and MdEVs normalized to the housekeeping gene *β-Actin*. *hCD81mNG* levels were not differently expressed between Exomap1$^{+/+}$ and AstroGreen$^{+/+}$ for both BdEVs and MdEVs. (C) Expression levels for *tdTomato* and *Iba1* as determined by qRT-PCR were significantly higher in Exomap1$^{+/+}$ compared to AstroGreen$^{+/+}$ in BdEVs (top). tdTomato was also significantly higher in Exomap1$^{+/+}$ compared to AstroGreen$^{+/+}$ MdEVs (bottom). No significant differences for *Iba1* in MdEVs were observed (bottom). Gene expression levels were normalized *to* the housekeeping gene *β-Actin*. Data represent three independent experiments and are presented as the mean with SEM (error bars). Multi-comparison one-way ANOVA, $*p < 0.05$, $**p < 0.01$, $****p < 0.0001$, not significant (n.s.)

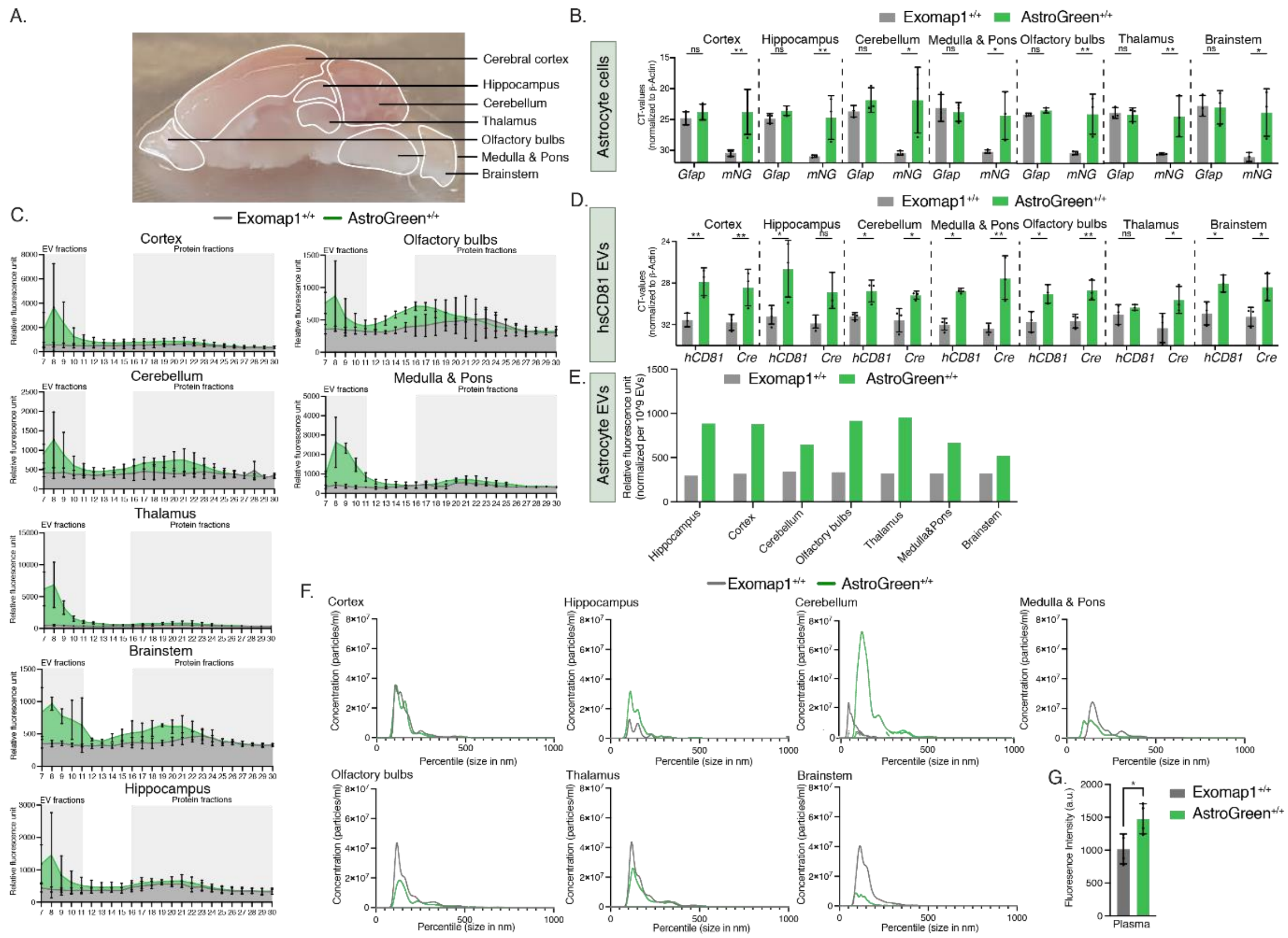

Figure S4. Astrocyte-derived extracellular vesicles isolated from specific brain regions.

(A) Image of a mouse brain showing one hemisphere sagittal dissected defining the olfactory bulbs, cortex, cerebellum, hippocampus, thalamus, medulla & pons and the brainstem. (B) qT-PCR analysis of cells isolated from different brain regions comparing AstroGreen$^{+/+}$ and Exomap1$^{+/+}$ transgenic mice brains. *Gfap* expression levels are equal between Exomap1$^{+/+}$ and AstroGreen$^{+/+}$-derived astrocyte cells, mNeonGreen (mNG) is only present in astrocytes isolated from the AstroGreen$^{+/+}$ brain areas. (C) Fluorescence for mNeonGreen (Excitation: 500 nm Emission: 520 nm) of each SEC fraction (7-30) were measured per brain region, showing mNeonGreen expression primarily in EV fractions 7-11. (D) EVs for each brain region were enriched for HsCD81 using microbeads to measure gene expression levels (*HsCD81mNG* and *Cre*) for both AstroGreen$^{+/+}$ and *Exomap1*$^{+/+}$ showing significant increased levels of *hCD81mNG* in all brain regions except the thalamus in AstroGreen$^{+/+}$-EVs. *Cre* was significantly higher expressed in each region in AstroGreen$^{+/+}$-EVs except in the hippocampus. Highest levels of hCD81 were observed in the hippocampus. (E) BdEV fractions 7-11 of each brain area (Cortex, Hippocampus, Cerebellum, Olfactory Bulbs, Thalamus, Medulla & Pons, Brainstem) were pooled and quantified

by NTA and normalized per 10^9 particle count (**Table 3**). (F) The number of EVs were normalized between brain regions and mNeonGreen fluorescence was measured to compare the relative fluorescence units (RFU) per 10^9 EVs among brain structures. (G) Plasma was isolated from whole blood of Exomap1$^{+/+}$ and AstroGreen$^{+/+}$ mice. Fluorescence intensity levels showed significant increased mNeonGreen in plasma derived from AstroGreen$^{+/+}$ mice compared to Exomap1$^{+/+}$ control. Data represent three independent experiments and are presented as the mean with SEM (error bars). Unpaired t test. *$p<0.05$, **$p<0.01$.

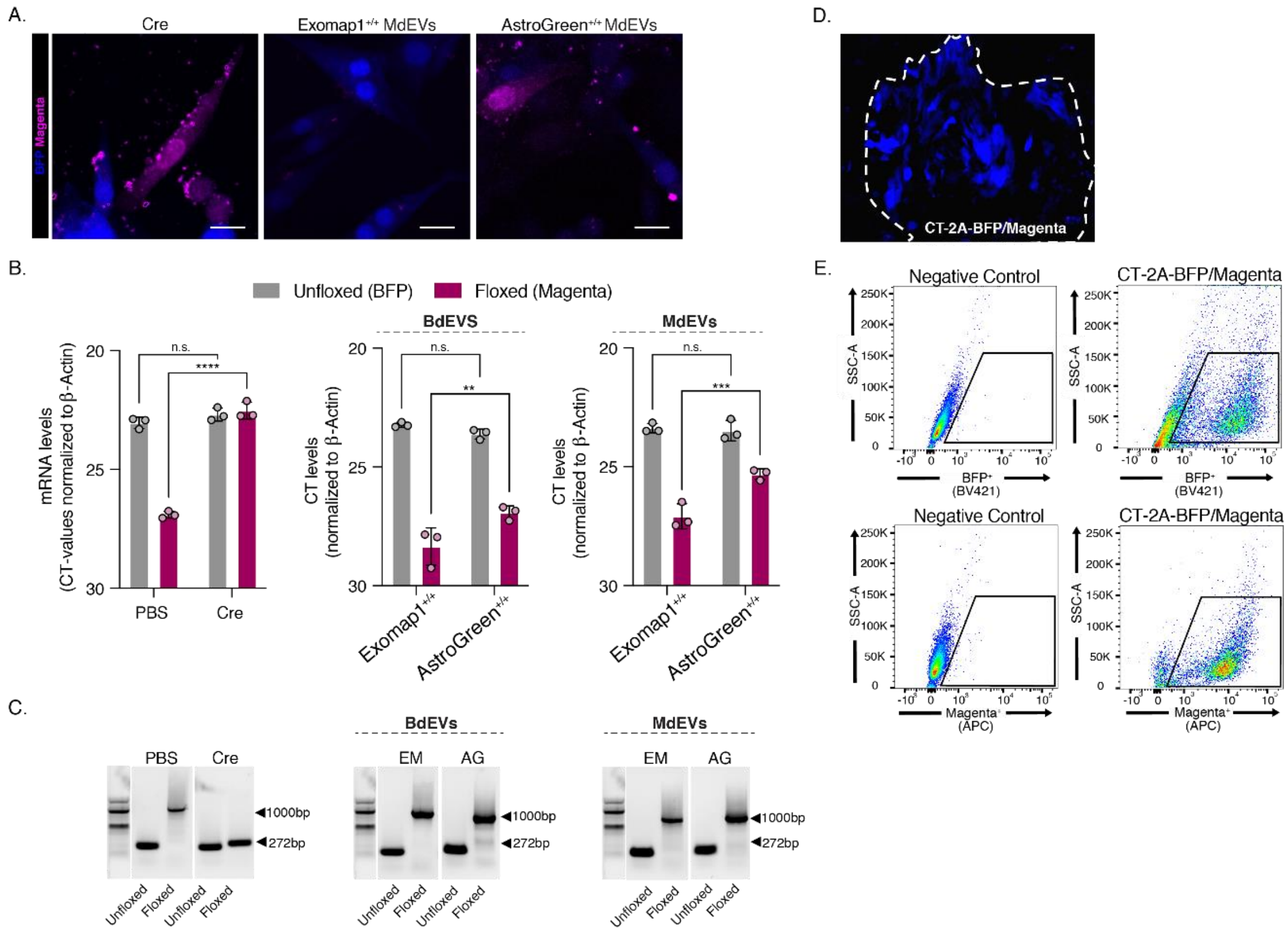

Figure S5. Tracking EV-mediated Cre and HsCD81 transfer in CT-2A-BFP-Magenta tumor. (A) Representative images of CT-2A-BFP/Magneta cells showing Magenta^{+} floxed cells post-transfection incubated for 5-days with AstroGreen$^{+/+}$ or Exomap1$^{+/+}$ MdEVs compared to the positive control cells transfected with 400 ng of the Cre expressing plasmid (Madisen et al., 2010). (Magnification 60x; scale bar 50 µm). (B) Expression levels of the unfloxed and floxed allele of the CT-2A-BFP cells *in vitro* were analyzed and we confirmed significantly increased expression levels of the floxed allele after Cre-transfection compared to PBS (left) or post-exposure of 5 days to AstroGreen$^{+/+}$ BdEVs (middle) or MdEVs (right) compared to Exomap1$^{+/+}$-derived EVs. Data represent three independent experiments and are presented as the mean with SEM (error bars). Multi-comparison one-way ANOVA, **p<0.01, *** p<0.001, ****<0.0001. (C) The floxed allele amplicon with a size of 272 bp was observed in CT-2A-BFP cells transfected with a Cre-expressing construct or when exposed to BdEVs or MdEVs. The 272 bp band was not observed in the PBS control of when cells were exposed to Exomap1$^{+/+}$-derived EVs. (D) CT-2A-BFP unfloxed tumor cells (blue) the tumor border highlighted as a white dotted line. 14-days post-implantation in the left striatum of a Exomap1$^{+/+}$ mouse. Data represent slices of one coronal

sectioned Exomap1$^{+/+}$ mouse brain. (Magnification 40x; scale bar 50 µm). (E) Representative flow cytometry plots show gating of the BFP (BV421) and Magenta (APC) populations of CT-2A cultured cells (Negative control) and the CT-2A-BFP/Magenta cell line to determine BFP^{+} unfloxed cells and Magenta^{+} floxed cells upon transfection with 400 ng of the plasmid expressing Cre. (F) Representative images of CT-2A tumor-bearing AstroGreen$^{+/+}$ brains at day 14 post-tumor implantation with mNeonGreen positive astrocytes shows HsCD81 (white) expression at the tumor border and inside the tumor. Tumor border highlighted with a red dashed line. (Magnification 40x; scale bar 50 µm).

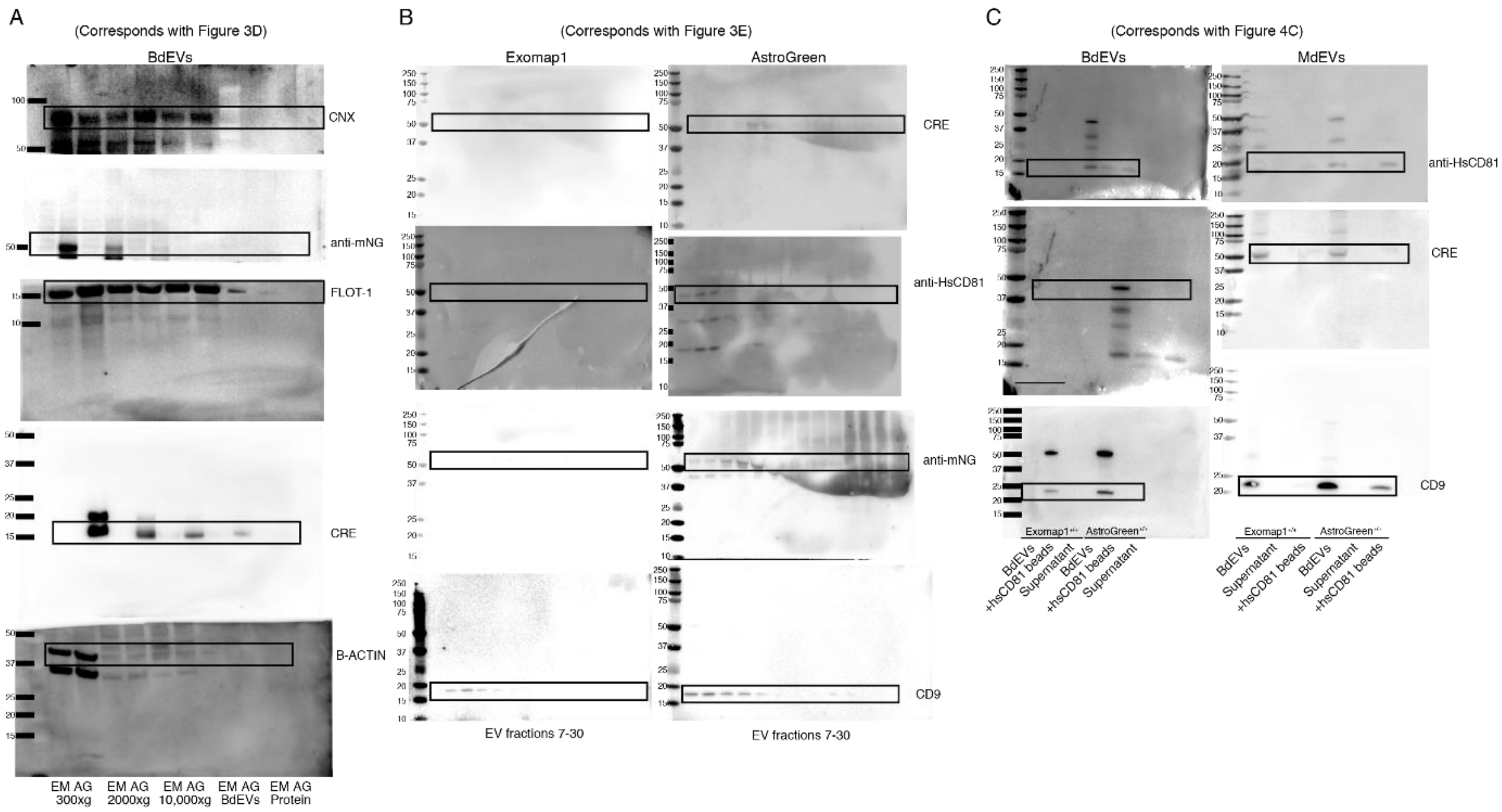

Figure S6. Images of western blot membranes.

(A-B) Representative images of raw western blot membranes corresponding to main figure 3D. (C) Raw western blot membranes corresponding to main figure 4D.

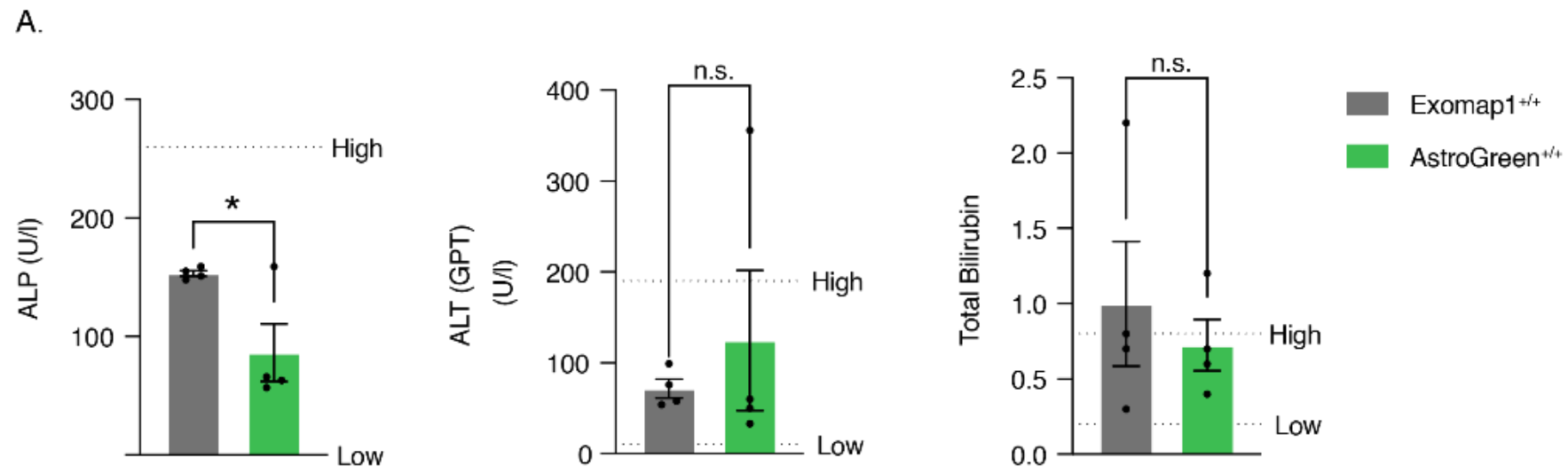

Figure S7. Blood analysis to evaluate systemic liver toxicity.

(A) Alkaline Phosphatase (ALP), Alanine transaminase (ALT) and total Bilirubin levels as measured in whole blood comparing ExoMap1$^{+/+}$ and AstroGreen$^{+/+}$ mice (n=4/group). Dotted lines marking low and high range in normal mice. Data is plotted as mean values with (SEM) error bars. Unpaired t test, ***<0.001, not significant (n.s.).
